# Supplementary material for: SorLA restricts TNFα release from microglia to shape a glioma-supportive brain microenvironment
Source: EMBO Rep. 2024 Mar 18;25(5):13. doi: 10.1038/s44319-024-00117-6 (PMC11094098; doi:10.1038/s44319-024-00117-6)
Supplement: Supplementary file 1 — Appendix [file 44319_2024_117_MOESM1_ESM.pdf]

Appendix

SorLA restricts TNFα release from microglia to shape glioma-supportive brain microenvironment

Paulina Kaminska, Peter L. Ovesen, Mateusz Jakiel, Tomasz Obrebski, Vanessa Schmidt, Michal Draminski, Aleksandra G. Bilska, Magdalena Bieniek, Jasper Anink, Bohdan Paterczyk, Anne Mette Gissel Jensen, Sylwia Piatek, Olav M. Andersen, Eleonora Aronica, Thomas E. Willnow, Bozena Kaminska, Michal J. Dabrowski, Anna R. Malik

Table of contents

Appendix Figure S1. Validation of *SORL1* expression pattern in GAMs in an independent dataset. 3

Appendix Figure S2. Raw data obtained for control genes in qPCR experiments. 5

Appendix Figure S3. Characterization of human iMG cells. 7

Appendix Figure S4. Interaction between SorLA and TNFα is abolished after increasing the salt concentration in co-IP procedure. 8

Appendix Figure S5. Loss of SorLA does not influence colocalization of TNFα with lysosomes, late endosomes and Golgi in primary murine microglia. 9

Appendix Figure S6. Cytokine levels in tumor-free and tumor-bearing hemispheres at 14 and 21 days post-implantation. 11

Appendix Figure S7. Loss of SorLA influences microglia exclusively in the tumor-bearing hemisphere. 12

Appendix Figure S8. Loss of SorLA has no impact on GAMs infiltration. 14

Appendix Figure S9. Hematological analysis of WT and SorLA-KO glioma-bearing mice does not reveal significant differences in the numbers of blood cells between the genotypes. 15

Appendix Table S1. Clinical characteristics of patients' GBM samples and the summary of SorLA immunoreactivity in Iba1+ cells. 16

Appendix Table S2. Proportions of *SORL1*- and *AIF1*-expressing cells in scRNA/snRNA-seq data from human GBM samples 18

Appendix Table S3. Expression data extracted from Szulzewsky et al., Plos One, 2015 (7) 19

Appendix Table S4. Expression levels of GAMs marker genes in all cell clusters in newly-diagnosed GBM from Abdelfattah et al. 20

Appendix Table S5. Expression levels of tumor cells marker genes in all cell clusters in newly-diagnosed GBM from Abdelfattah et al. 21

Appendix Table S6. Expression levels of lymphocytes marker genes in all cell clusters in newly-diagnosed GBM from Abdelfattah et al. 22

Appendix Table S7. Expression levels of oligodendrocytes, smooth muscle cells and endothelial cells marker genes in all cell clusters in newly-diagnosed GBM from Abdelfattah et al. 23

Appendix Table S8. Expression levels of GAMs marker genes in all cell clusters in primary GBM from Neftel et al. 24

Appendix Table S9. SorLA domains covered by mini-receptors used in the study. 24

Appendix References 25

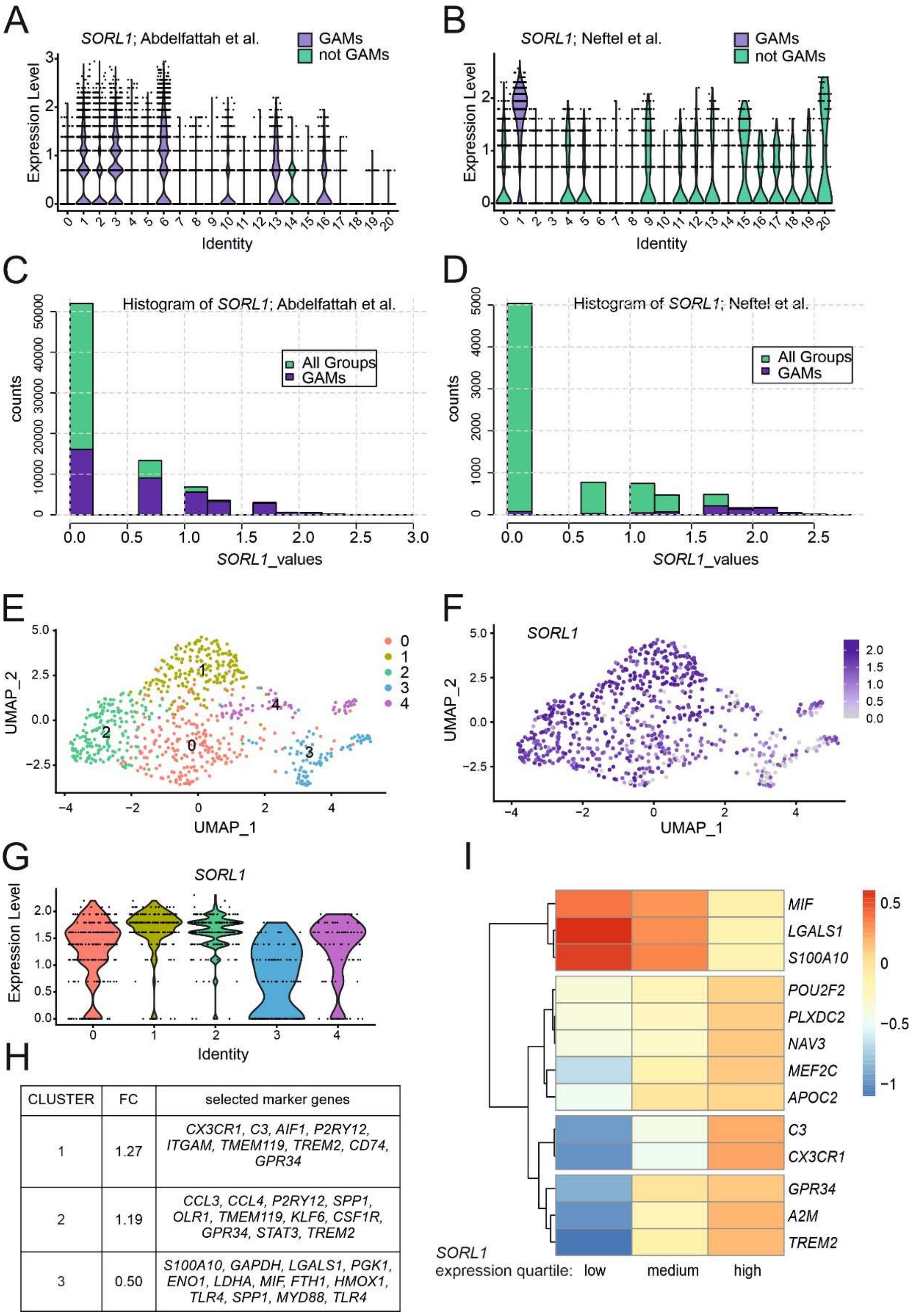

CLUSTER	FC	selected marker genes
1	1.27	*CX3CR1, C3, AIF1, P2RY12, ITGAM, TMEM119, TREM2, CD74, GPR34*
2	1.19	*CCL3, CCL4, P2RY12, SPP1, OLR1, TMEM119, KLF6, CSF1R, GPR34, STAT3, TREM2*
3	0.50	*S100A10, GAPDH, LGALS1, PGK1, ENO1, LDHA, MIF, FTH1, HMOX1, TLR4, SPP1, MYD88, TLR4*

Appendix Figure S1. Validation of *SORL1* expression pattern in GAMs in an independent dataset. (A-B) Violin plots illustrating the expression of *SORL1* in all clusters present in the ndGBM data from Abdelfattah et al. (A) and primary GBM data from Neftel at al. (B). Purple color denotes clusters containing GAMs and green denotes those without GAMs. Highest *SORL1* expression is seen in clusters containing GAMs in both datasets.

(C-D) Histograms showing distribution of *SORL1* expression in all cells and in GAMs in two datasets: ndGBM from Abdelfattah et al. (C) and primary GBM from Neftel et al. (D). Cell counts are depicted on y-axis.

(E) UMAP projection of single cells showing human GAMs from GBM tumors described in Neftel et al., grouped in 5 clusters.

(F) UMAP plot showing *SORL1* expression levels normalized with SCT, in clusters of human GAMs selected from Neftel et al.

(G) Violin plot illustrating the expression of *SORL1* in 5 clusters of GAMs from Neftel et al., as exemplified in (E). Cluster 3 shows the lowest expression of *SORL1* among GAMs.

(H) Selected marker genes of GAMs clusters with highest and lowest *SORL1* expression levels. FC – *SORL1* expression fold change; mean cluster expression against mean expression in remaining clusters. Related to Figure 1C.

(I) Heatmap showing expression levels of selected genes (mean values of standardized gene expression data) in GAMs from Neftel et al., as exemplified in (E), in the context of discretized values of *SORL1* gene expression. Related to Figure 1D.

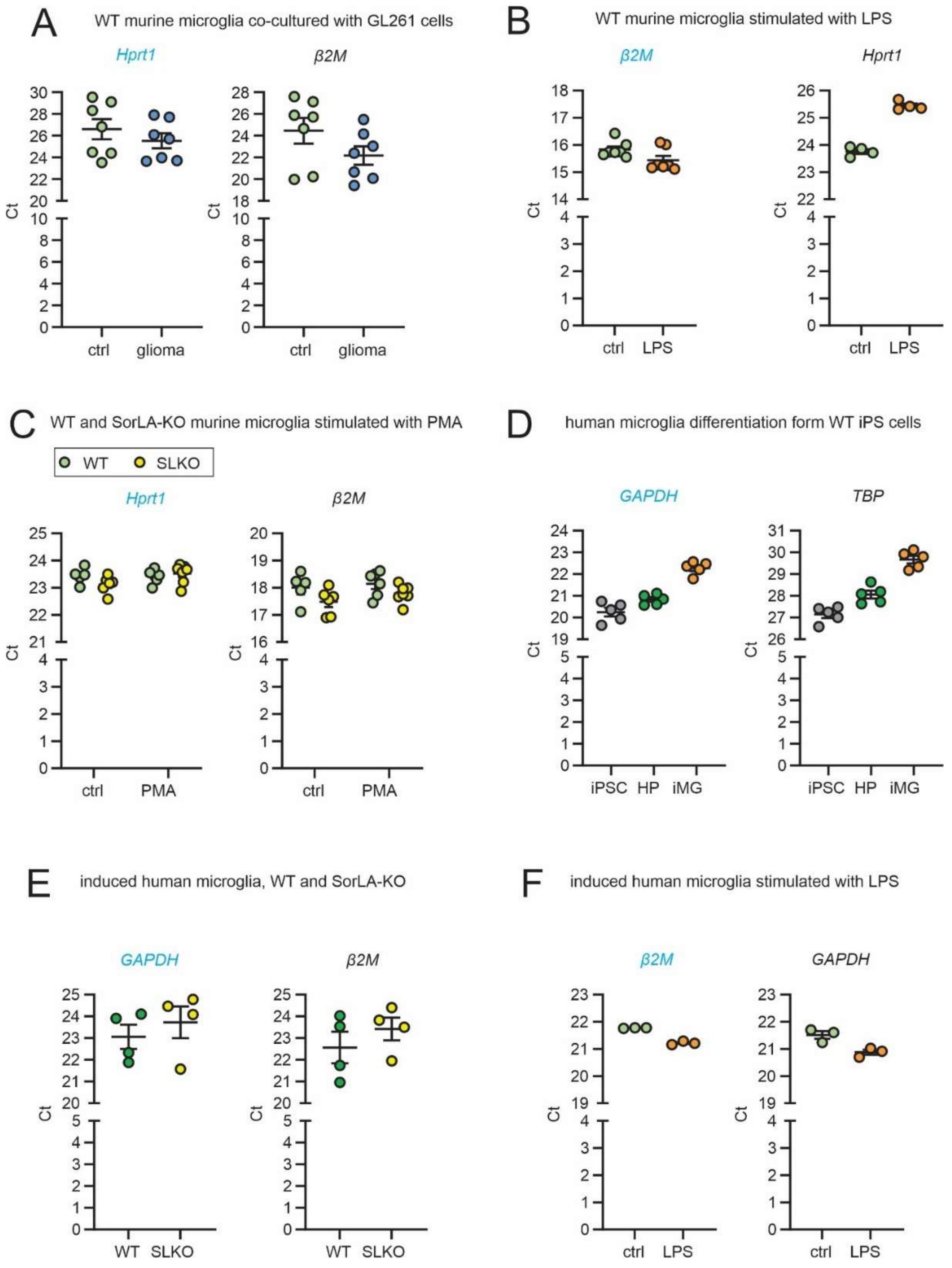

Appendix Figure S2. Raw data obtained for control genes in qPCR experiments.

Comparison of Ct values for indicated control genes performed in order to assess their suitability as reference genes in qPCR experiments. Genes selected as internal controls for data quantification presented in the manuscript are highlighted in blue.

(A) Primary murine microglial cells were co-cultured with GL261 glioma cells. Related to Figure 3A., left panel.

(B) Primary murine microglial cells were stimulated with LPS. Related to Figure 3A., right panel.

(C) Primary murine microglial cells, wild-type and SorLA-KO, were stimulated with PMA. Related to Figure 3C.

(D) Human iPS cells (iPSC) were differentiated to generate microglia. HP, hematopoietic progenitors; iMG, induced microglia. Related to Figure 3F.

(E) iMG cells were generated from WT and SorLA-KO human iPS cells. Related to Figure S3B.

(F) WT iMG cells were stimulated with LPS. Related to Figure 3H.

Data information: In (A-F) data are presented as mean ± SEM. In F, error bar for β2M is too small to be displayed. No statistical analysis was performed as we did not intend to directly compare Ct values obtained for particular genes under experimental conditions.

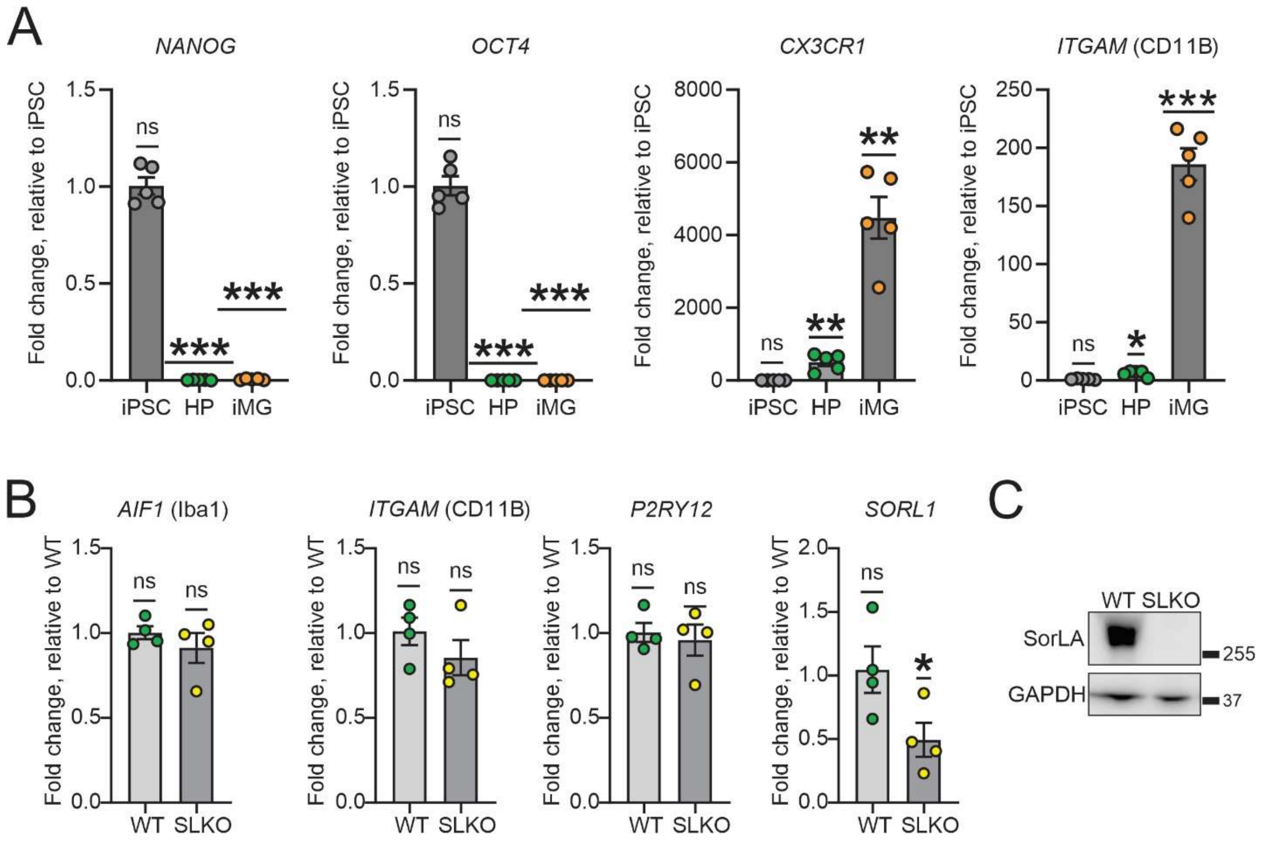

Appendix Figure S3. Characterization of human iMG cells.

(A) Expression levels of marker genes for pluripotent stem cells (*NANOG*, *OCT4*) and microglia (*CX3XR1*, *ITGAM*) in iPSCs, HPs and iMG during microglia differentiation as assessed by qRT-PCR (relative to *GAPDH*). n=5 biological replicates.

(B) Expression levels of marker genes for microglia (*AIF1*, *ITGAM*, *P2RY12*) and *SORL1* in WT and SorLA-KO (SLKO) iMG as assessed by qRT-PCR (relative to *GAPDH*). n=4 biological replicates.

(C) Representative result of western blot analysis detecting SorLA in WT and SorLA-KO (SLKO) iMG. GAPDH serves as a loading control.

Data information: In (A-B) data are presented as mean ± SEM. ns, not significant; *, $p<0.05$; **, $p < 0.01$; ***, $p < 0.001$ in one sample t-test compared to 1.

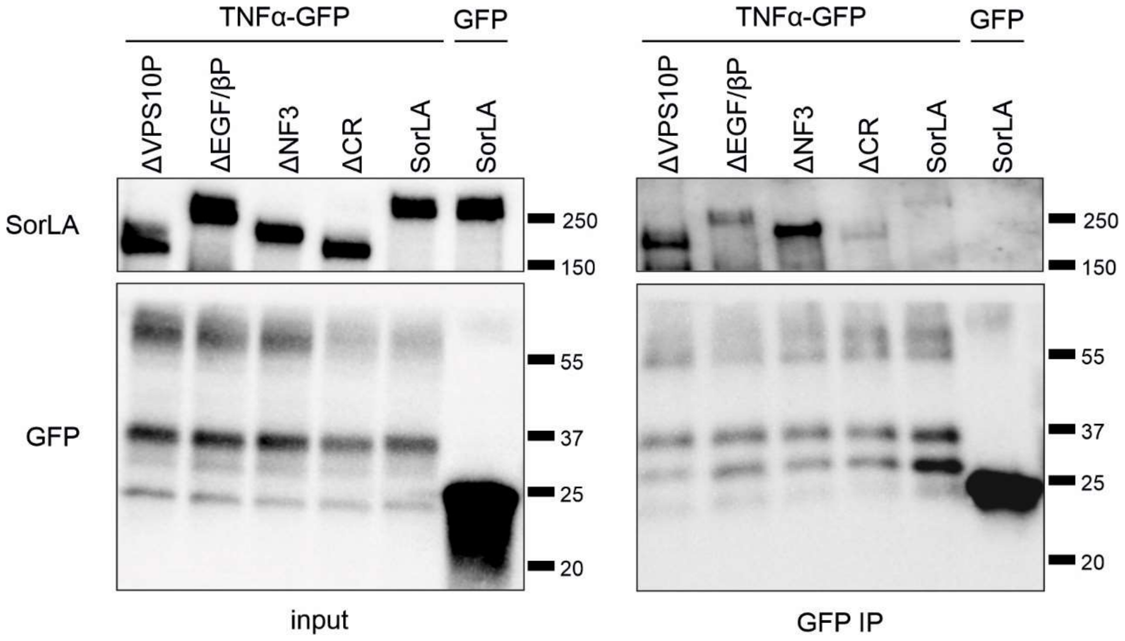

Appendix Figure S4. Interaction between SorLA and TNFα is abolished after increasing the salt concentration in co-IP procedure.

Co-immunoprecipitation of SorLA deletion mutants with TNFα-GFP overexpressed in HEK293 cells (as in Figure EV4B) after GFP-IP performed in increased salt concentration (300 mM NaCl instead of 150 mM NaCl). GFP serves as a negative control.

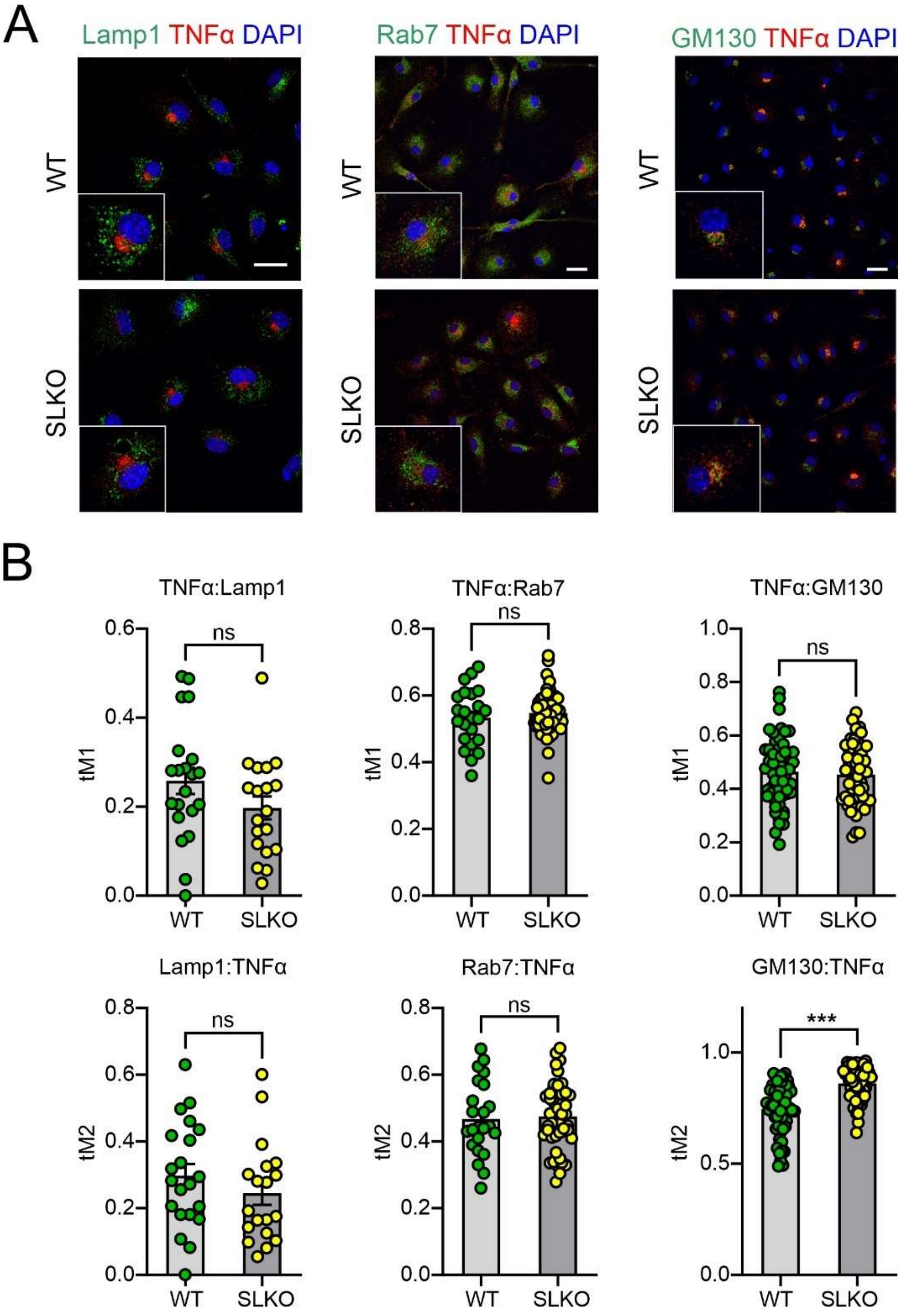

Appendix Figure S5. Loss of SorLA does not influence colocalization of TNFα with lysosomes, late endosomes and Golgi in primary murine microglia.

(A) Representative images of primary murine WT and SorLA-KO (SLKO) microglia stimulated with PMA for 24h, immunostained for TNFα and the markers of lysosomes (Lamp1), late endosomes (Rab7) and Golgi (GM130). Cells were counterstained with DAPI. Scale bars, 20 µm.

(B) Thresholded Manders coefficients (tM) calculated for colocalization of TNFα with the markers of subcellular compartments as exemplified in (A). n=19-21 cells for Lamp1, n=36-48 cells for Rab7, n=56 cells for GM130.

Data information: Data in (B) are presented as mean ± SEM. ns, not significant; ***, $p < 0.001$ in unpaired two-tailed t-test.

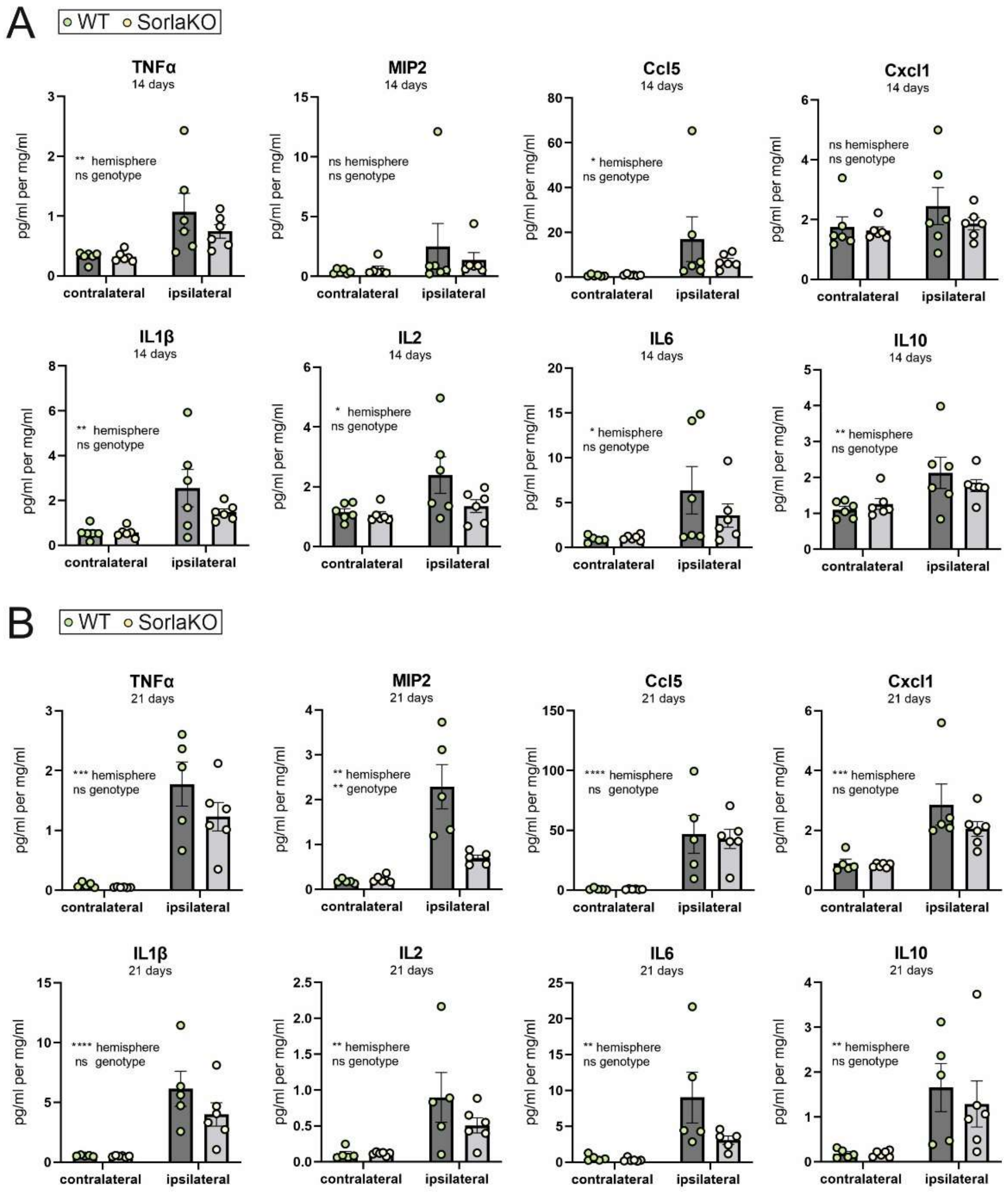

Appendix Figure S6. Cytokine levels in tumor-free and tumor-bearing hemispheres at 14 and 21 days post-implantation.

Cytokine levels were measured with multiplex ELISA in the protein lysates derived from tumor-free (contralateral) and tumor-bearing (ipsilateral) hemispheres of WT and SorLA-KO mice (A) 14 and (B) 21 days after implantation of GL261 tdTomato+Luc+ cells. Cytokines levels were normalized to total protein content. n= 5-6 mice per genotype.

Data information: Data are presented as mean ± SEM. ns, not significant; *, $p < .05$; **, $p < .01$; ***, $p < .001$; ****; $p<0.0001$ in two-way ANOVA.

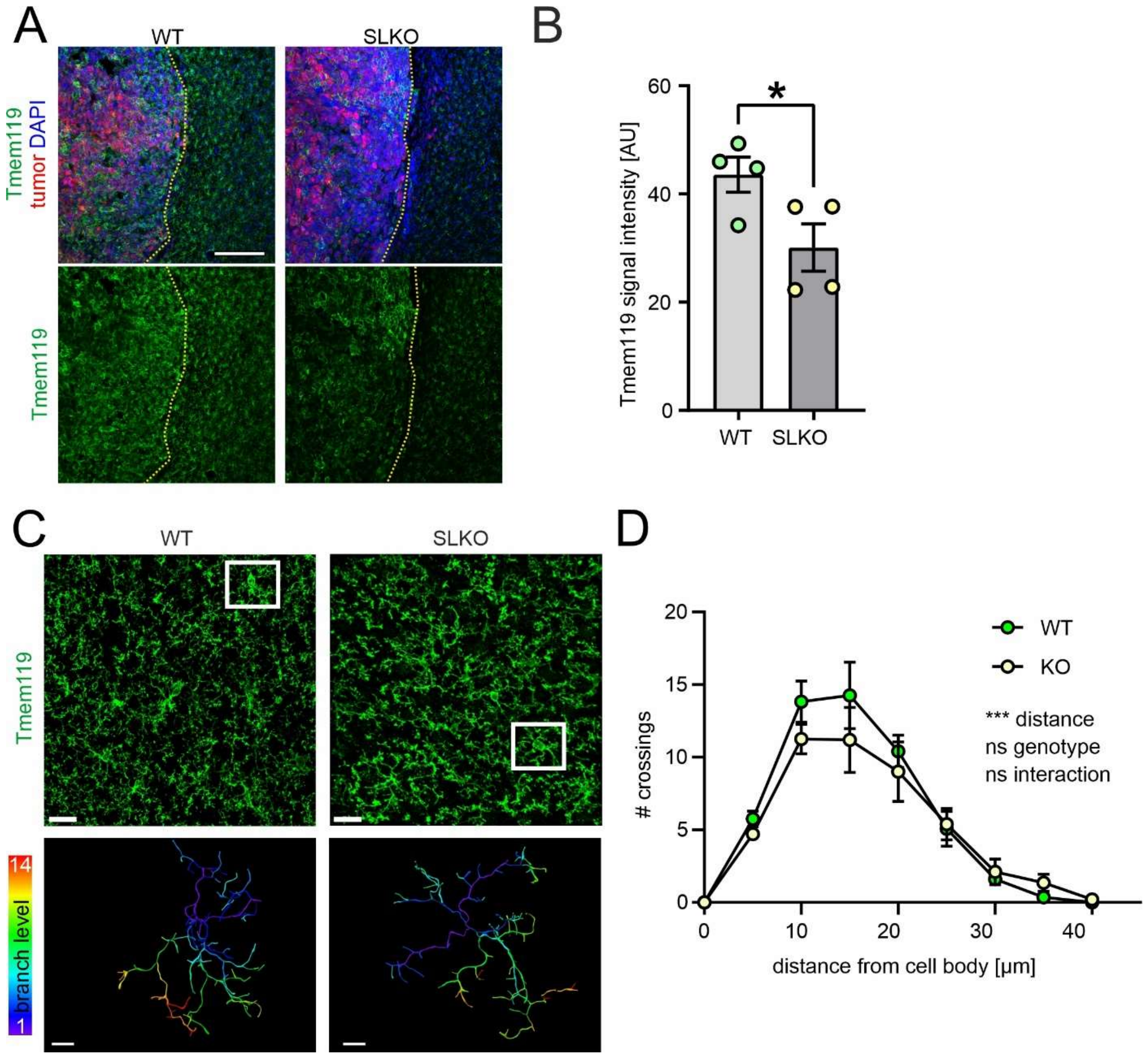

Appendix Figure S7. Loss of SorLA influences microglia exclusively in the tumor-bearing hemisphere.

(A) Representative images of the sections from glioma-bearing WT and SorLA-KO brains 21 days after implantation of GL261-tdTomato+Luc+ cells, immunostained for the microglia marker, Tmem119. Tumor cells are seen in red. Yellow dotted line marks tumor border. Sections were counterstained with DAPI (blue). Scale bar, 200 µm.

(B) Mean signal intensity for Tmem119 (quantified in the area outside the tumor). n=4 mice per genotype.

(C) Upper panel: representative images of microglia morphology revealed by Tmem119 staining in WT and SorLA-KO mice in the contralateral hemispheres of glioma-bearing brains 21 days post-implantation. Scale bar, 20 µm. White box indicates the cell reconstructed below. Lower panel shows reconstructed microglia branches; color depicts branch level. Scale bar, 5µm.

(D) Sholl analysis of microglia morphology reconstructed as in (C). n=4 mice per genotype; for each mouse, 4-5 cells were quantified and an average of obtained values was treated as an individual data point.

Data information: In (B,D) data are represented as mean ± SEM. ns, not significant; *, $p<0.05$; ***, $p<0.001$ in unpaired two-tailed t-test (B) or two-way ANOVA (D).

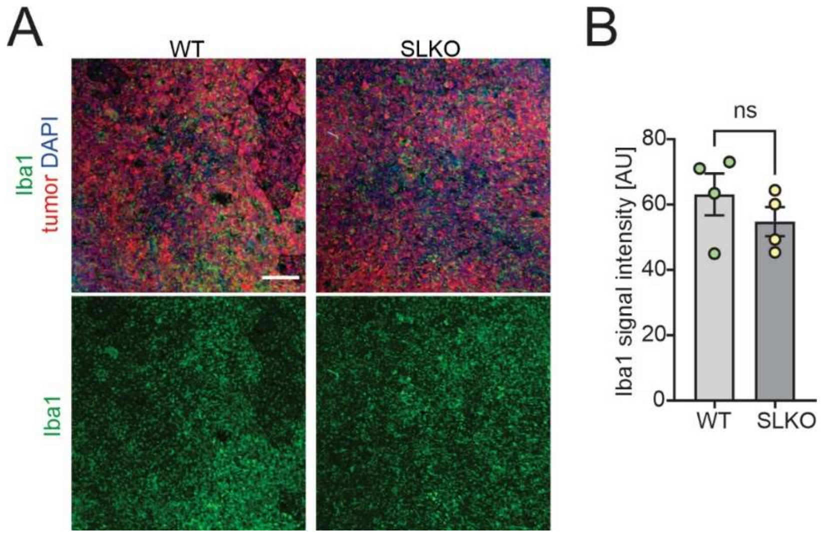

Appendix Figure S8. Loss of SorLA has no impact on GAMs infiltration.

(A) Representative images of the sections from glioma-bearing WT and SorLA-KO brains 21 days after implantation of GL261-tdTomato+Luc+ cells, immunostained for a marker of microglia/macrophages, Iba1. Tumor cells are seen in red. Sections were counterstained with DAPI (blue). Scale bar, 200 µm. **(B)** Mean signal intensity for Iba1 (quantified in the area inside the tumor). n=4 mice per genotype.

Data information: In (B) data are represented as mean ± SEM. ns, not significant in unpaired two-tailed t-test.

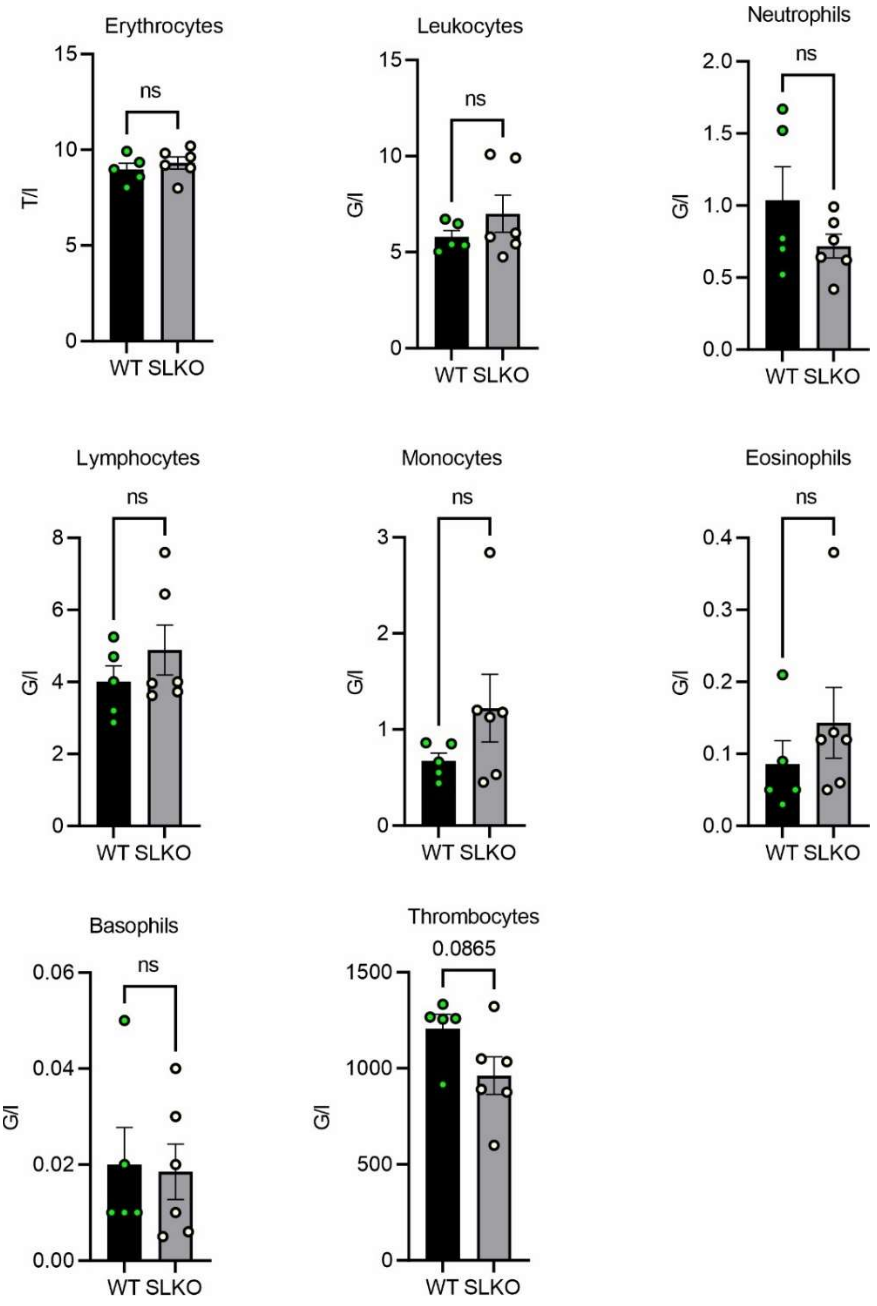

Appendix Figure S9. Hematological analysis of WT and SorLA-KO glioma-bearing mice does not reveal significant differences in the numbers of blood cells between the genotypes.

Erythrocytes, leukocytes, neutrophils, lymphocytes, monocytes, eosinophils, basophils and thrombocytes counts measured in the peripheral blood of WT and SorLA-KO glioma-bearing animals 21 days after implantation. n=5-6 mice per genotype.

Data information: Data are presented as mean ± SEM. ns, not significant in unpaired two-tailed t-test. For thrombocytes, p-value is shown.

Appendix Table S1. Clinical characteristics of patients' GBM samples and the summary of SorLA immunoreactivity in Iba1+ cells.

Patient #	Age, gender	tumor location	Pathology/diagnosis	SorLA expression in Iba1+ cells
1	43, male	Parietal R	Astrocytoma IDH-wildtype, CNS WHO grade 3	+
2	63, male	Frontal R	Glioblastoma, IDH-wildtype, CNS WHO grade 4.	-
3	11, female	Frontal L	Astrocytoma, IDH1-mutant, WHO CNS grade 4	+
4	56, male	Frontal R	Glioblastoma, IDH-wildtype, CNS WHO grade 4	+
5	17, male	Frontal R	Glioblastoma, IDH-wildtype, CNS WHO grade 4	Hard to interpret
6	16, male	Parietal R	Glioblastoma, IDH-wildtype, CNS WHO grade 4	-
7	64, male	Temporal R	Glioblastoma, IDH-wildtype, CNS WHO grade 4	-
8	50, male	Temporal R	Glioblastoma, IDH-wildtype, CNS WHO grade 4	-
9	49, female	Frontal R	Glioblastoma, IDH-wildtype, CNS WHO grade 4	-
10	54, female	Cerebellum	Glioblastoma, IDH-wildtype, CNS WHO grade 4	+
11	63, female	Parietal L	Glioblastoma, IDH-wildtype, CNS WHO grade 4	+
12	74, male	Fronto-parietal L	Glioblastoma, IDH-wildtype, CNS WHO grade	+
13	48, male	Parieto-temporal L	Glioblastoma, IDH-wildtype, CNS WHO grade 4	-
14	11, male	Occipital L	Glioblastoma, IDH-wildtype, CNS WHO grade 4	-

15	40, male	Frontal L	Glioblastoma, IDH-wildtype, CNS WHO grade 4	Hard to interpret

Appendix Table S2. Proportions of *SORL1*- and *AIF1*-expressing cells in scRNA/snRNA-seq data from human GBM samples.

Reference	Total number of cells	% *AIF1+*	% *SORL1+*	% *SORL1+* in *AIF1+* population	Comments	Dataset
Abdelfattah et al. (1)	80 157	52.4	35.1	53.1	selection of ndGBM samples from this dataset	GSE182109
Pombo Antunes et al. (2)	21 154	97	55.6	56.7	CD45+ sorted cells	GSE163120
Chen et al. (3)	15 247	7.8	5.8	17.2	combined datasets	GSE141383
Neftel et al. (4)	7 894	9.4	36.2	87.3	sorted immune and non-immune cells	GSE131928
Sankowski et al. (5)	568	58.8	93.0	97.0	CD45+ sorted cells	GSE135437
Wang et al. (6)	44 295	0.6	12.8	42.7	single nuclei RNAseq; selection of ndGBM samples from this dataset	GSE174554

% *AIF1+*, %*SORL1+* - percentage of all cells where each of these genes was expressed; 100% is the set of all cells.

% *SORL1+* in *AIF1+* population, percentage of *AIF1*-expressing cells that also expressed *SORL1*; 100% is the set of cells where *AIF1* was expressed.

Appendix Table S3. Expression data extracted from Szulzewsky et al., Plos One, 2015 (7)

glioma-associated vs naive CD11b+ cells					
transcript	AffyID	LogFC	FC	p.value	Adj.p.Val
Sorcs2	10529515	-1.0137488	0.4952577	2.13E-07	2.63E-05
Sorl1	10592535	1.67434829	3.1917514	0.000794346	0.007875

Appendix Table S4. Expression levels of GAMs marker genes in all cell clusters in newly-diagnosed GBM from Abdelfattah et al.

cluster	***AIF1***	***CD68***	***ITGAM***	***P2RY12***	***TMEM119***	***CX3CR1***	**GAMs score (Σ pct.1 values for all GAMs marker genes)**
0	n.a.	n.a.	n.a.	n.a.	n.a.	n.a.	n.a.
1	0.929	0.800	n.a.	n.a.	0.588	n.a.	2.317
2	0.690	0.551	n.a.	0.725	0.540	0.59	3.096
3	0.821	0.708	n.a.	n.a.	n.a.	n.a.	1.529
4	n.a.	n.a.	n.a.	n.a.	n.a.	n.a.	n.a.
5	n.a.	n.a.	n.a.	n.a.	n.a.	n.a.	n.a.
6	n.a.	n.a.	n.a.	0.507	0.556	0.589	1.652
7	n.a.	n.a.	n.a.	n.a.	n.a.	n.a.	n.a.
8	n.a.	n.a.	n.a.	n.a.	n.a.	n.a.	n.a.
9	n.a.	n.a.	n.a.	n.a.	n.a.	n.a.	n.a.
10	0.731	0.674	0.569	n.a.	n.a.	n.a.	1.974
11	n.a.	n.a.	n.a.	n.a.	n.a.	n.a.	n.a.
12	n.a.	n.a.	n.a.	n.a.	n.a.	n.a.	n.a.
13	0.887	0.787	0.612	n.a.	0.796	n.a.	3.082
14	n.a.	n.a.	n.a.	n.a.	n.a.	n.a.	n.a.
15	n.a.	n.a.	n.a.	n.a.	n.a.	n.a.	n.a.
16	0.823	n.a.	n.a.	n.a.	n.a.	n.a.	0.823
17	n.a.	n.a.	n.a.	n.a.	n.a.	n.a.	n.a.
18	n.a.	n.a.	n.a.	n.a.	n.a.	n.a.	n.a.
19	n.a.	n.a.	n.a.	n.a.	n.a.	n.a.	n.a.
20	n.a.	n.a.	n.a.	n.a.	n.a.	n.a.	n.a.

pct.1 values for chosen marker genes in clusters 0-20 in ndGBM; data from Abdelfattah et al., Nat Commun, 2022 (1)

n.a. - given gene is not a marker gene for the particular cluster

Clusters 1, 2, 3, 6, 10, 13, 16 were classified as GAMs-containing clusters; these cells were pooled for further analysis of GAMs.

Appendix Table S5. Expression levels of tumor cells marker genes in all cell clusters in newly-diagnosed GBM from Abdelfattah et al.

cluster	***CDK4***	***MT1X***	***ATRX***	***CCND2***	***MDM2***	***SOX4***	***CD9***	***CDK6***	***S100B***	**Tumor score (Σ pct.1 values for all marker genes)**
0	0.528	n.a.	n.a.	0.759	n.a.	0.639	0.749	0.735	0.810	4.220
1	n.a.	n.a.	n.a.	n.a.	n.a.	n.a.	n.a.	n.a.	n.a.	n.a.
2	n.a.	n.a.	n.a.	n.a.	n.a.	n.a.	n.a.	n.a.	n.a.	n.a.
3	n.a.	n.a.	n.a.	n.a.	n.a.	n.a.	n.a.	n.a.	n.a.	n.a.
4	n.a.	n.a.	n.a.	n.a.	n.a.	n.a.	n.a.	n.a.	n.a.	n.a.
5	n.a.	n.a.	n.a.	n.a.	n.a.	n.a.	n.a.	n.a.	n.a.	n.a.
6	n.a.	n.a.	n.a.	n.a.	n.a.	n.a.	n.a.	n.a.	n.a.	n.a.
7	n.a.	0.864	n.a.	n.a.	0.562	n.a.	0.718	n.a.	n.a.	2.144
8	n.a.	0.598	n.a.	n.a.	n.a.	n.a.	0.767	n.a.	0.754	2.119
9	0.667	n.a.	n.a.	0.747	n.a.	n.a.	0.764	0.730	n.a.	2.908
10	n.a.	n.a.	n.a.	n.a.	n.a.	n.a.	n.a.	n.a.	n.a.	n.a.
11	n.a.	n.a.	n.a.	n.a.	n.a.	n.a.	n.a.	n.a.	n.a.	n.a.
12	n.a.	n.a.	n.a.	n.a.	n.a.	n.a.	0.793	n.a.	0.950	1.743
13	n.a.	n.a.	n.a.	n.a.	n.a.	n.a.	n.a.	n.a.	n.a.	n.a.
14	n.a.	0.533	n.a.	n.a.	n.a.	n.a.	n.a.	n.a.	n.a.	0.533
15	n.a.	n.a.	0.631	0.504	n.a.	0.989	n.a.	0.711	n.a.	2.835
16	n.a.	n.a.	n.a.	n.a.	n.a.	n.a.	n.a.	n.a.	n.a.	n.a.
17	n.a.	n.a.	n.a.	n.a.	n.a.	n.a.	n.a.	n.a.	n.a.	n.a.
18	n.a.	n.a.	n.a.	n.a.	n.a.	n.a.	n.a.	n.a.	n.a.	n.a.
19	n.a.	n.a.	n.a.	n.a.	n.a.	n.a.	n.a.	n.a.	n.a.	n.a.
20	n.a.	n.a.	n.a.	n.a.	n.a.	0.706	0.804	n.a.	n.a.	1.51

pct.1 values for chosen marker genes in clusters 0-20 in ndGBM; data from Abdelfattah et al., Nat Commun, 2022 (1)

n.a. - given gene is not a marker gene for the particular cluster

Clusters 0, 7, 8, 9, 15 were classified as tumor cells-containing clusters; these cells were pooled for further analysis.

Appendix Table S6. Expression levels of lymphocytes marker genes in all cell clusters in newly-diagnosed GBM from Abdelfattah et al.

cluster	***GZMK***	***CD3E***	***PTPRC***	***CCL5***	***IL32***	***CD69***	***CD52***	**Lymphocytes score (Σ pct.1 values for all marker genes)**
0	n.a.	n.a.	n.a.	n.a.	n.a.	n.a.	n.a.	n.a.
1	n.a.	n.a.	n.a.	n.a.	n.a.	n.a.	n.a.	n.a.
2	n.a.	n.a.	n.a.	n.a.	n.a.	n.a.	n.a.	n.a.
3	n.a.	n.a.	n.a.	n.a.	n.a.	n.a.	n.a.	n.a.
4	n.a.	n.a.	n.a.	n.a.	n.a.	n.a.	n.a.	n.a.
5	0.721	0.710	0.849	0.771	0.801	0.860	0.856	5.568
6	n.a.	n.a.	0.556	n.a.	n.a.	n.a.	n.a.	0.556
7	n.a.	n.a.	n.a.	n.a.	n.a.	n.a.	n.a.	n.a.
8	n.a.	n.a.	n.a.	n.a.	n.a.	n.a.	n.a.	n.a.
9	n.a.	n.a.	n.a.	n.a.	n.a.	n.a.	n.a.	n.a.
10	n.a.	n.a.	n.a.	n.a.	n.a.	n.a.	n.a.	n.a.
11	n.a.	n.a.	n.a.	n.a.	n.a.	n.a.	n.a.	n.a.
12	n.a.	n.a.	n.a.	n.a.	n.a.	n.a.	n.a.	n.a.
13	n.a.	n.a.	n.a.	n.a.	n.a.	n.a.	n.a.	n.a.
14	n.a.	n.a.	n.a.	n.a.	n.a.	n.a.	n.a.	n.a.
15	n.a.	n.a.	n.a.	n.a.	n.a.	n.a.	n.a.	n.a.
16	n.a.	n.a.	0.77	n.a.	n.a.	n.a.	n.a.	0.77
17	0.853	0.882	0.882	0.831	0.824	0.893	0.812	5.977
18	n.a.	n.a.	n.a.	n.a.	n.a.	n.a.	n.a.	n.a.
19	0.761	0.866	0.881	0.731	0.925	0.836	0.948	5.082
20	n.a.	n.a.	n.a.	n.a.	n.a.	1	1	2

pct.1 values for chosen marker genes in clusters 0-20 in ndGBM; data from Abdelfattah et al. Nat Commun. 2022 (1)

n.a. - given gene is not a marker gene for the particular cluster

Clusters 5, 17, 19 were classified as lymphocytes-containing clusters; these cells were pooled for further analysis.

Appendix Table S7. Expression levels of oligodendrocytes, smooth muscle cells and endothelial cells marker genes in all cell clusters in newly-diagnosed GBM from Abdelfattah et al.

	**Oligodendrocytes**			**Smooth muscle cells**			**Endothelial cells**			
cluster	***MBP***	***CNP***	**Σ**	***ACTA2***	***TAGLN***	**Σ**	***EDN1***	***PECAM1***	***ANGPT2***	**Σ**
0	n.a.	n.a.	n.a.	n.a.	n.a.	n.a.	n.a.	n.a.	n.a.	n.a.
1	n.a.	n.a.	n.a.	n.a.	n.a.	n.a.	n.a.	n.a.	n.a.	n.a.
2	n.a.	n.a.	n.a.	n.a.	n.a.	n.a.	n.a.	n.a.	n.a.	n.a.
3	n.a.	n.a.	n.a.	n.a.	n.a.	n.a.	n.a.	n.a.	n.a.	n.a.
4	n.a.	n.a.	n.a.	n.a.	n.a.	n.a.	n.a.	n.a.	n.a.	n.a.
5	n.a.	n.a.	n.a.	n.a.	n.a.	n.a.	n.a.	n.a.	n.a.	n.a.
6	n.a.	n.a.	n.a.	n.a.	n.a.	n.a.	n.a.	n.a.	n.a.	n.a.
7	n.a.	n.a.	n.a.	n.a.	n.a.	n.a.	n.a.	n.a.	n.a.	n.a.
8	n.a.	n.a.	n.a.	n.a.	n.a.	n.a.	n.a.	n.a.	n.a.	n.a.
9	n.a.	n.a.	n.a.	n.a.	n.a.	n.a.	n.a.	n.a.	n.a.	n.a.
10	n.a.	n.a.	n.a.	n.a.	n.a.	n.a.	n.a.	n.a.	n.a.	n.a.
11	n.a.	n.a.	n.a.	0.816	0.889	1.705	n.a.	n.a.	n.a.	n.a.
12	0.996	0.959	1.955	n.a.	n.a.	n.a.	n.a.	n.a.	n.a.	n.a.
13	n.a.	n.a.	n.a.	n.a.	n.a.	n.a.	n.a.	0.664	n.a.	0.664
14	n.a.	n.a.	n.a.	n.a.	n.a.	n.a.	0.926	0.684	0.633	2.243
15	n.a.	n.a.	n.a.	n.a.	n.a.	n.a.	n.a.	n.a.	n.a.	n.a.
16	n.a.	n.a.	n.a.	n.a.	n.a.	n.a.	n.a.	n.a.	n.a.	n.a.
17	n.a.	n.a.	n.a.	n.a.	n.a.	n.a.	n.a.	n.a.	n.a.	n.a.
18	n.a.	n.a.	n.a.	n.a.	n.a.	n.a.	n.a.	n.a.	n.a.	n.a.
19	n.a.	n.a.	n.a.	n.a.	n.a.	n.a.	n.a.	n.a.	n.a.	n.a.
20	n.a.	n.a.	n.a.	n.a.	n.a.	n.a.	n.a.	n.a.	n.a.	n.a.

pct.1 values for chosen marker genes in clusters 0-20 in ndGBM; data from Abdelfattah et al., Nat Commun. 2022 (1)

n.a. - given gene is not a marker gene for the particular cluster

Cluster 11 was classified as smooth muscle cells-containing cluster; cluster 12 was classified as oligodendrocytes-containing cluster; cluster 14 was classified as endothelial cells-containing cluster.

Appendix Table S8. Expression levels of GAMs marker genes in all cell clusters in primary GBM from Neftel et al.

cluster	*AIF1*	*CD68*	*ITGAM*	*P2RY12*	*TMEM119*	*CX3CR1*	**GAMs score (Σ pct.1 values for all GAMs marker genes)**
0	n.a.	n.a.	n.a.	n.a.	n.a.	n.a.	n.a.
1	0,93	0,989	0,747	0,592	0,58	0,726	4,564
2 - 20	n.a.	n.a.	n.a.	n.a.	n.a.	n.a.	n.a.

pct.1 values for chosen marker genes in clusters 0-20 in data from Neftel et al., 2019 (4)

n.a. - given gene is not a marker gene for the particular cluster

Cluster 1 was classified as GAMs-containing cluster

Appendix Table S9. SorLA domains covered by mini-receptors used in the study.

**SORLA domains**	**Amino acid number according to UniProtKB: Q92673.2**
Signal peptide	1-28
propeptide	29-78
Furin cleavage site	79-82
VPS10P	124-755
EGF/β-propeller	756-1072
CR	1073-1550
FN3	1551-2136
transmembrane and cytoplasmic domain	2138-2158

Appendix References

1. N. Abdelfattah, *et al.*, Single-cell analysis of human glioma and immune cells identifies S100A4 as an immunotherapy target. *Nat Commun* **13**, 767 (2022).

2. A. R. Pombo Antunes, *et al.*, Single-cell profiling of myeloid cells in glioblastoma across species and disease stage reveals macrophage competition and specialization. *Nat Neurosci* **24**, 595–610 (2021).

3. A. X. Chen, *et al.*, Single-cell characterization of macrophages in glioblastoma reveals MARCO as a mesenchymal pro-tumor marker. *Genome Medicine* **13**, 88 (2021).

4. C. Neftel, *et al.*, An Integrative Model of Cellular States, Plasticity, and Genetics for Glioblastoma. *Cell* **178**, 835-849.e21 (2019).

5. R. Sankowski, *et al.*, Mapping microglia states in the human brain through the integration of high-dimensional techniques. *Nat Neurosci* **22**, 2098–2110 (2019).

6. L. Wang, *et al.*, A single-cell atlas of glioblastoma evolution under therapy reveals cell-intrinsic and cell-extrinsic therapeutic targets. *Nat Cancer* **3**, 1534–1552 (2022).

7. F. Szulzewsky. *et al.*, Glioma-associated microglia/macrophages display an expression profile different from M1 and M2 polarization and highly express Gpnmb and Spp1. *PLoS One* **10**. e0116644 (2015).
