# Supplementary material for: SorLA restricts TNFα release from microglia to shape a glioma-supportive brain microenvironment
Source: EMBO Rep. 2024 Mar 18;25(5):13. doi: 10.1038/s44319-024-00117-6 (PMC11094098; doi:10.1038/s44319-024-00117-6)
Supplement: Supplementary file 8 — Source Data Fig. 3 [file 44319_2024_117_MOESM8_ESM.zip › Figure 3/3G/3G README.docx]

The images were cropped prior to placing on the figure.
