# Supplementary figures and images for: SorLA restricts TNFα release from microglia to shape a glioma-supportive brain microenvironment
Source: EMBO Rep. 2024 Mar 18;25(5):13. doi: 10.1038/s44319-024-00117-6 (PMC11094098; doi:10.1038/s44319-024-00117-6)

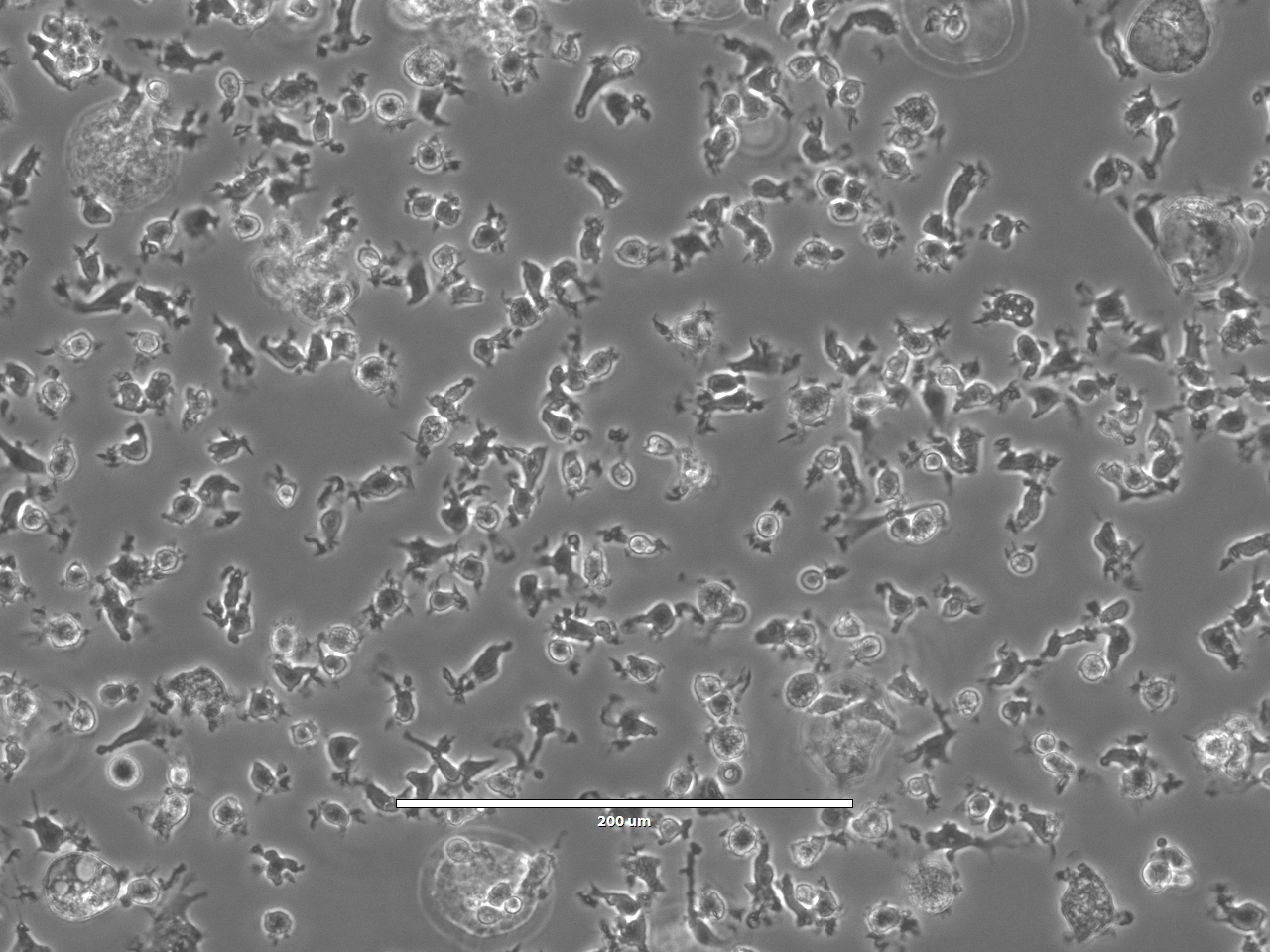

Supplement: Supplementary file 8 — Source Data Fig. 3 [file 44319_2024_117_MOESM8_ESM.zip › Figure 3/3E/3E iMG d38.tif]

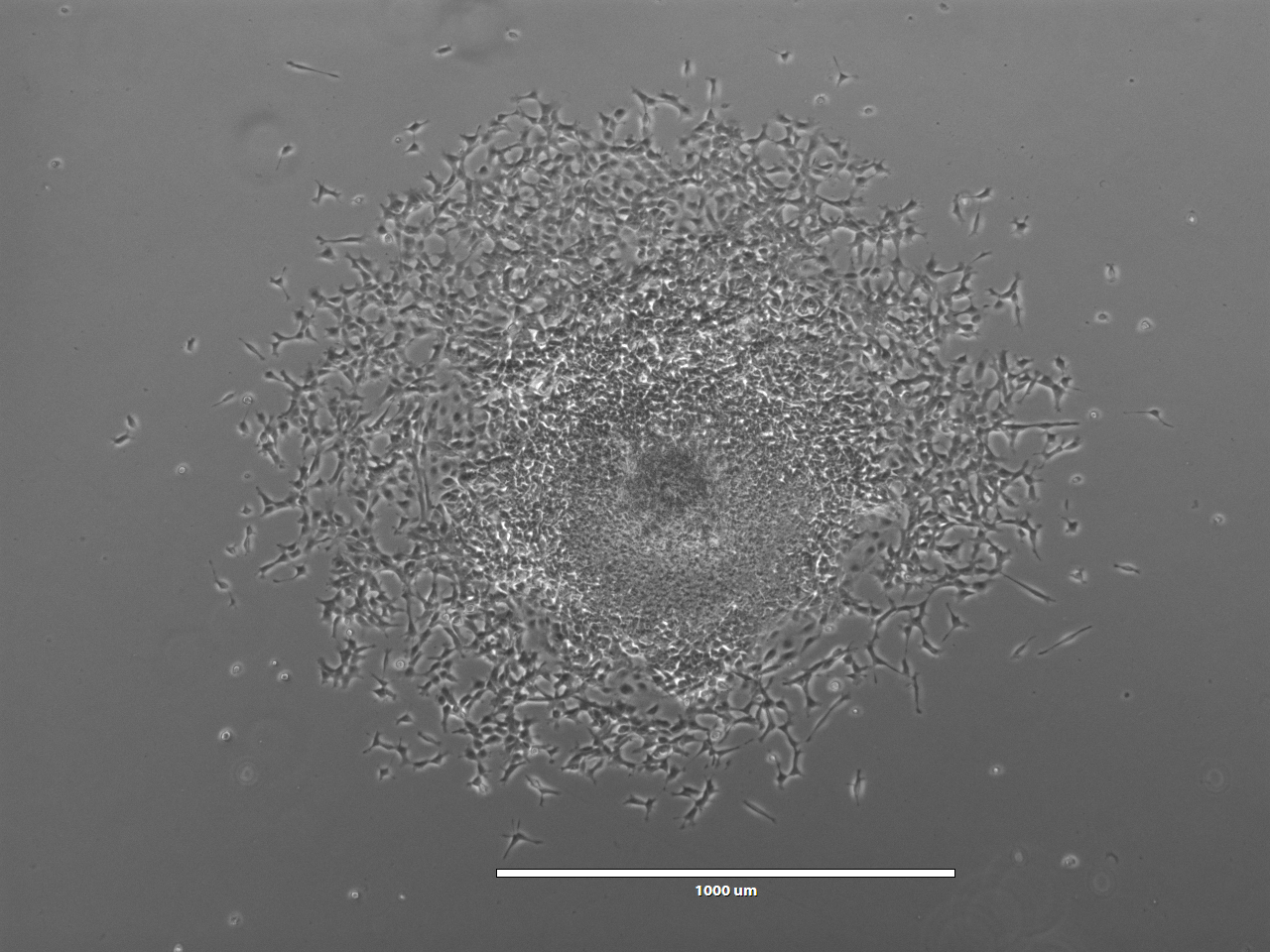

Supplement: Supplementary file 8 — Source Data Fig. 3 [file 44319_2024_117_MOESM8_ESM.zip › Figure 3/3E/3E HP d3.tif]

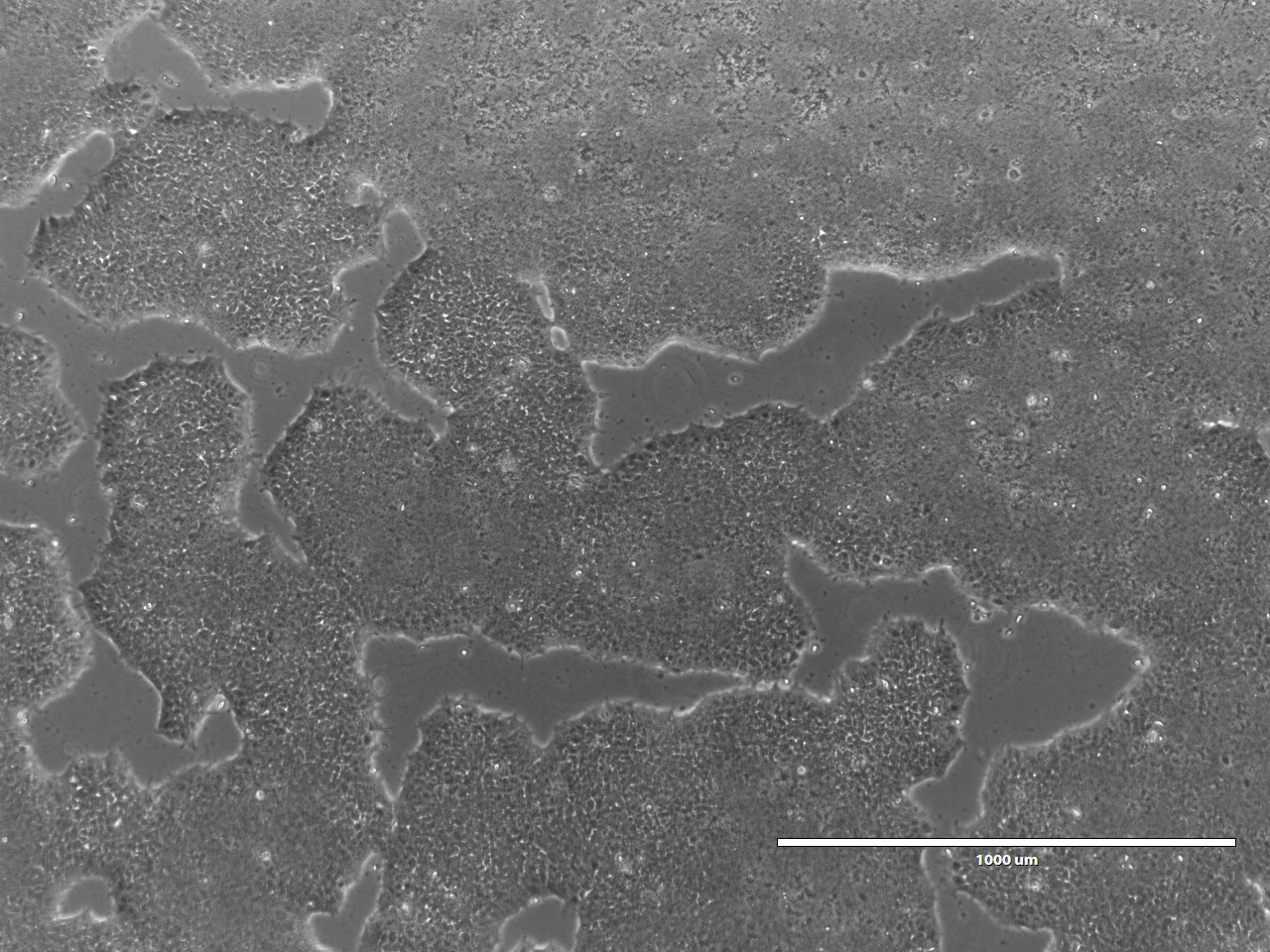

Supplement: Supplementary file 8 — Source Data Fig. 3 [file 44319_2024_117_MOESM8_ESM.zip › Figure 3/3E/3E iPSC d-1.tif]

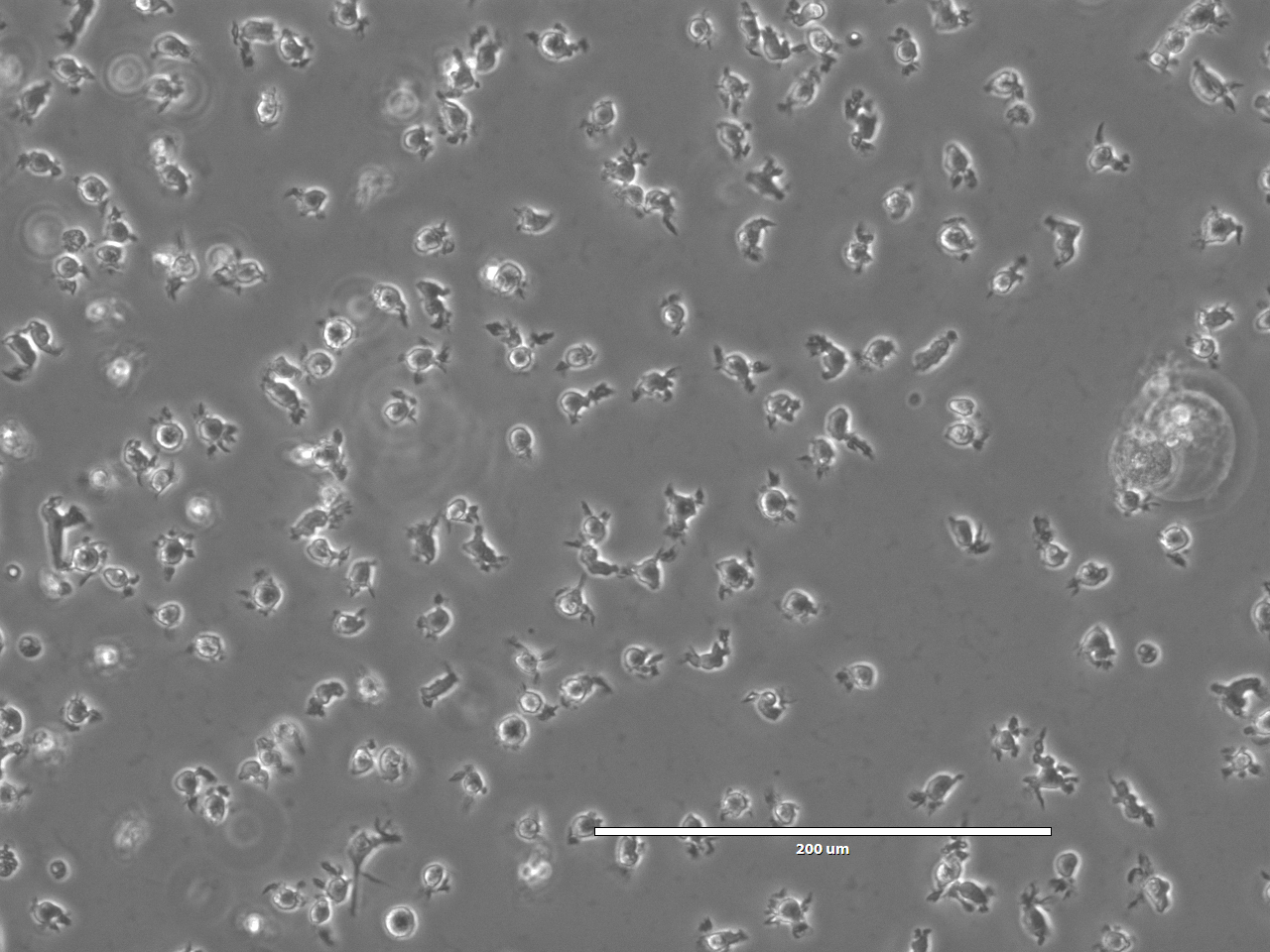

Supplement: Supplementary file 8 — Source Data Fig. 3 [file 44319_2024_117_MOESM8_ESM.zip › Figure 3/3E/3E iMG d23.tif]

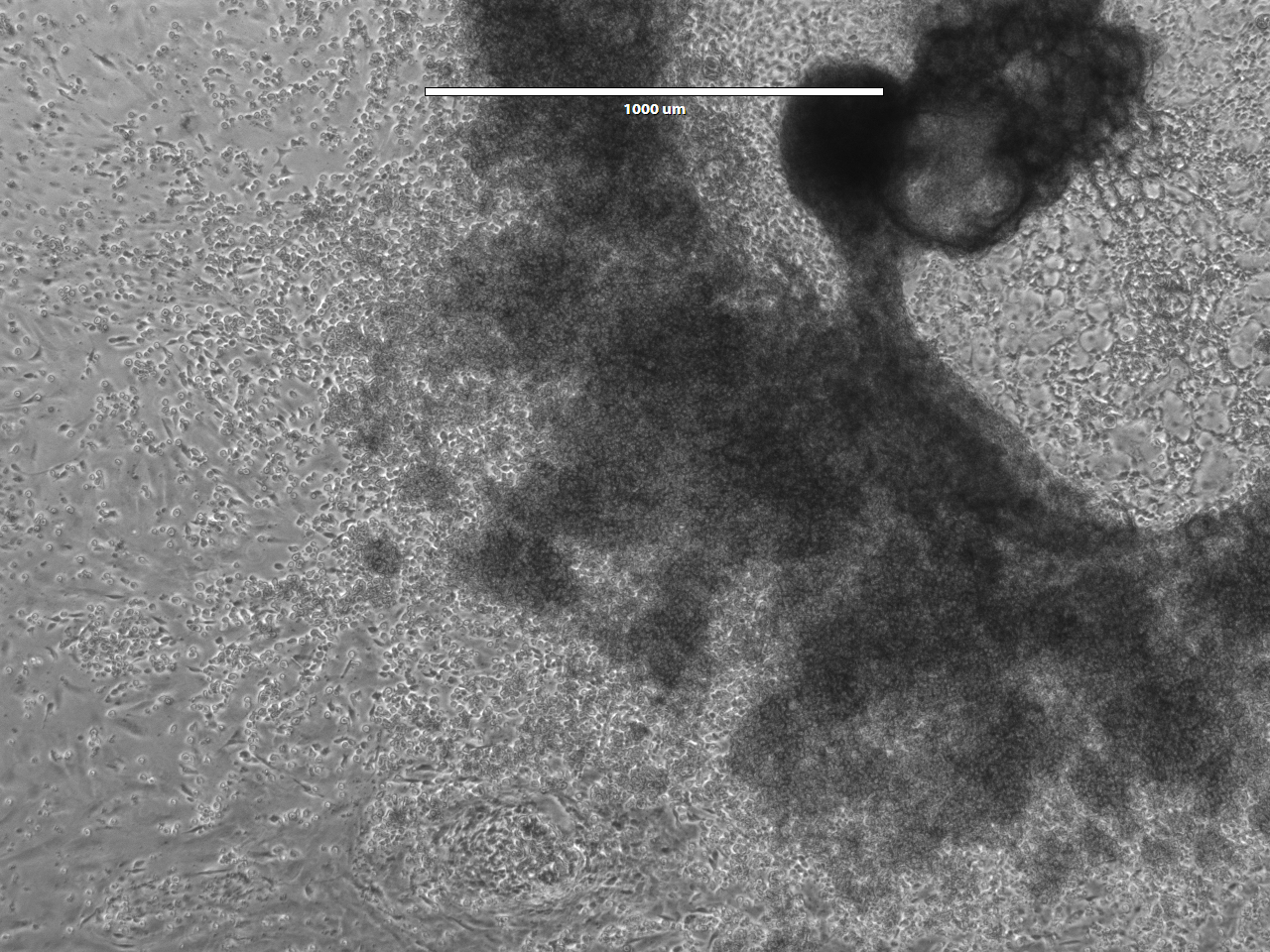

Supplement: Supplementary file 8 — Source Data Fig. 3 [file 44319_2024_117_MOESM8_ESM.zip › Figure 3/3E/3E HP d11.tif]

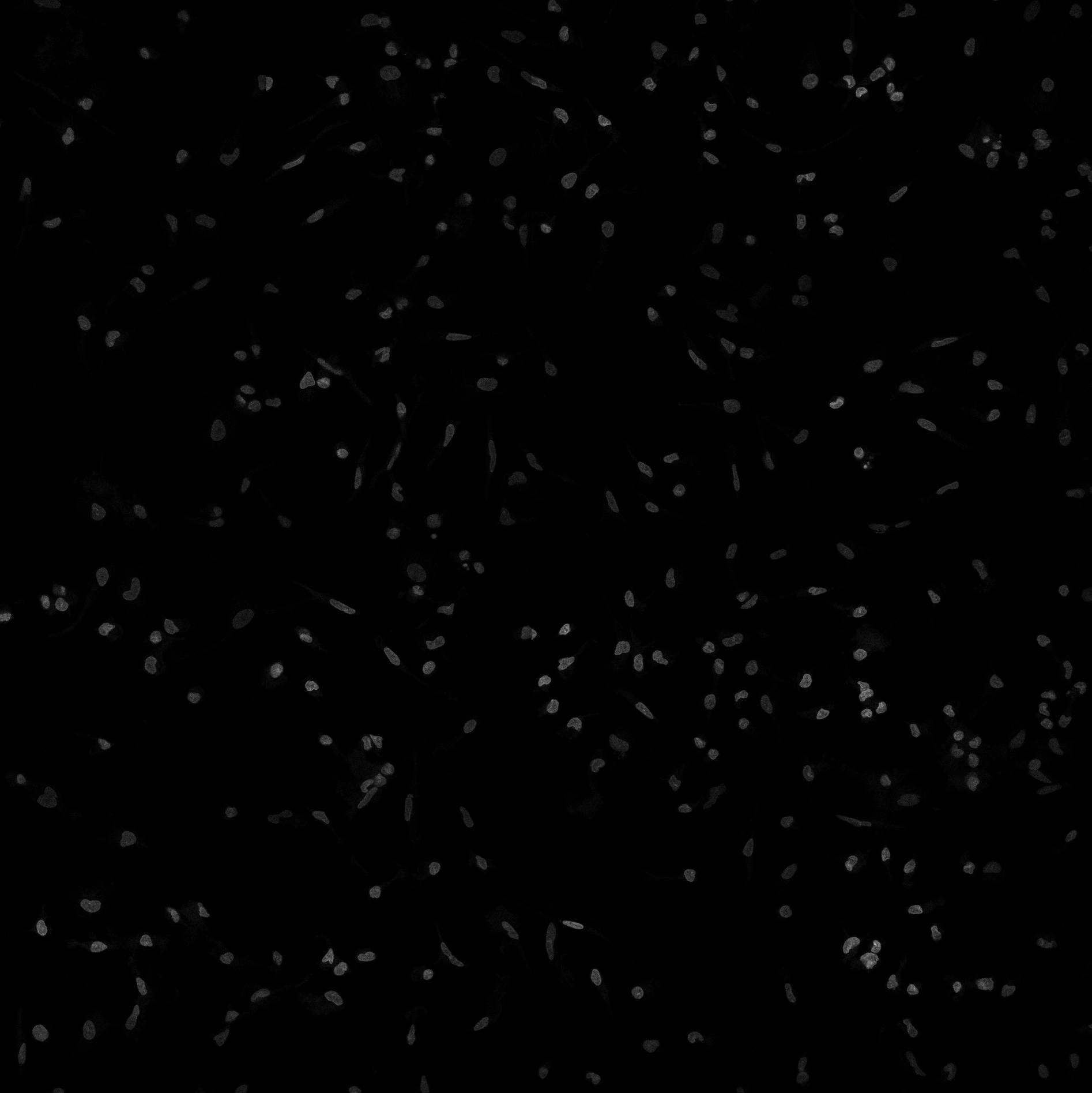

Supplement: Supplementary file 8 — Source Data Fig. 3 [file 44319_2024_117_MOESM8_ESM.zip › Figure 3/3G/iMG_DAPI_P2RY12_IBA1.tif]

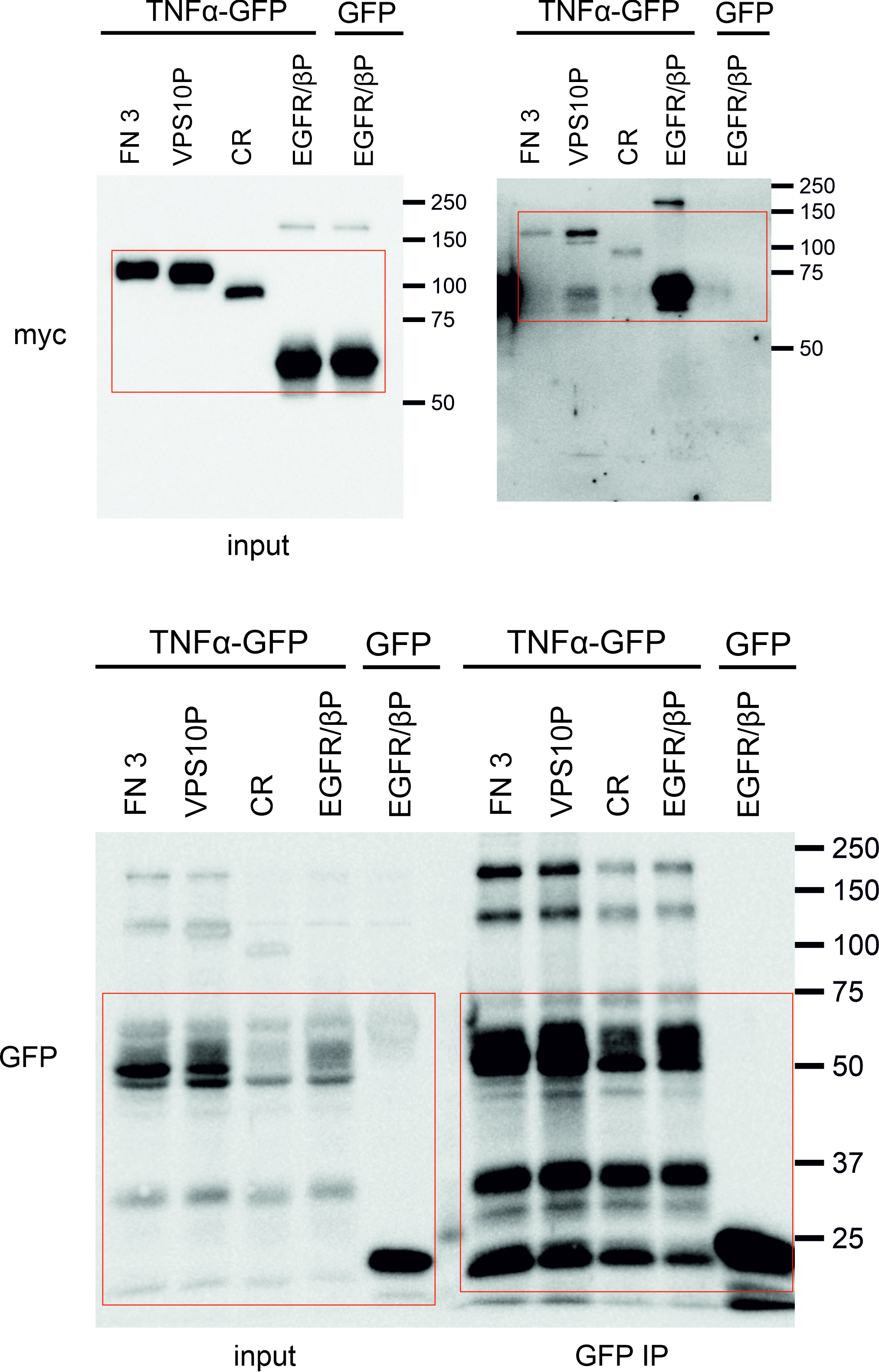

Supplement: Supplementary file 9 — Source Data Fig. 4 [file 44319_2024_117_MOESM9_ESM.zip › Figure 4/4E/4E coIP mini SorLA TNFa-GFP blots .tif]

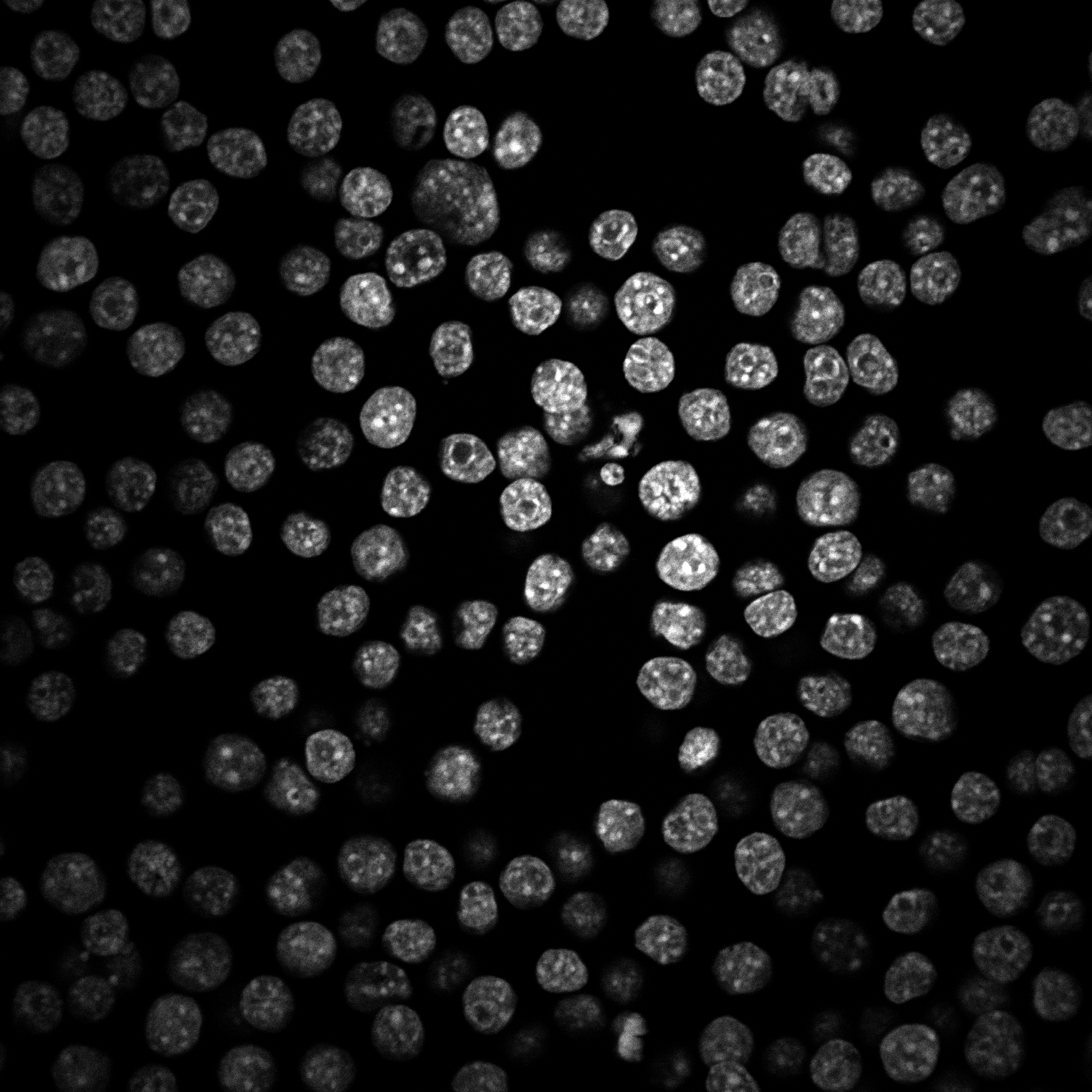

Supplement: Supplementary file 9 — Source Data Fig. 4 [file 44319_2024_117_MOESM9_ESM.zip › Figure 4/4A/4A BV2 PMAstim DAPI SORLA TNFa.tif]

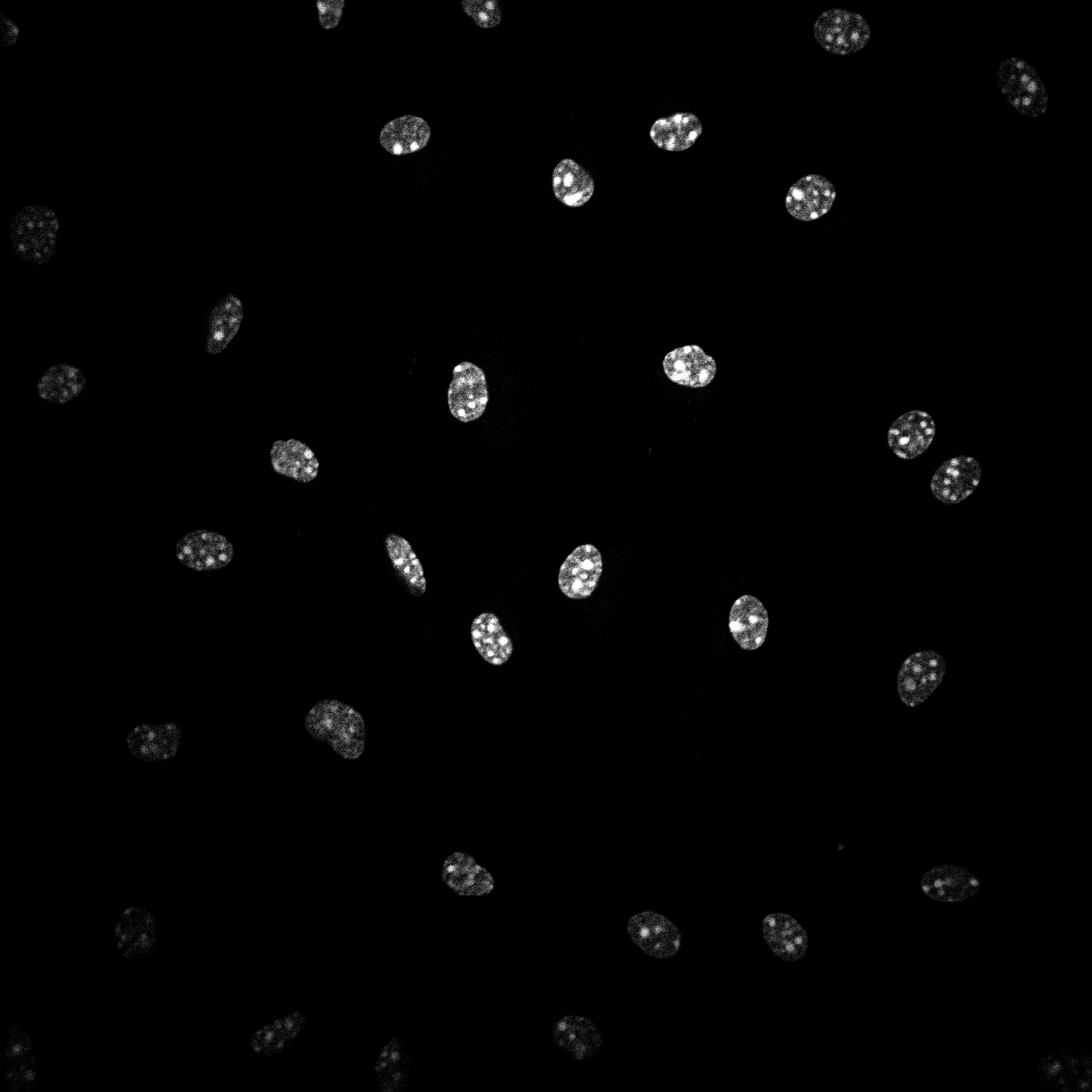

Supplement: Supplementary file 9 — Source Data Fig. 4 [file 44319_2024_117_MOESM9_ESM.zip › Figure 4/4G/4G WT PMAstim DAPI RAB11 TNF.tif]

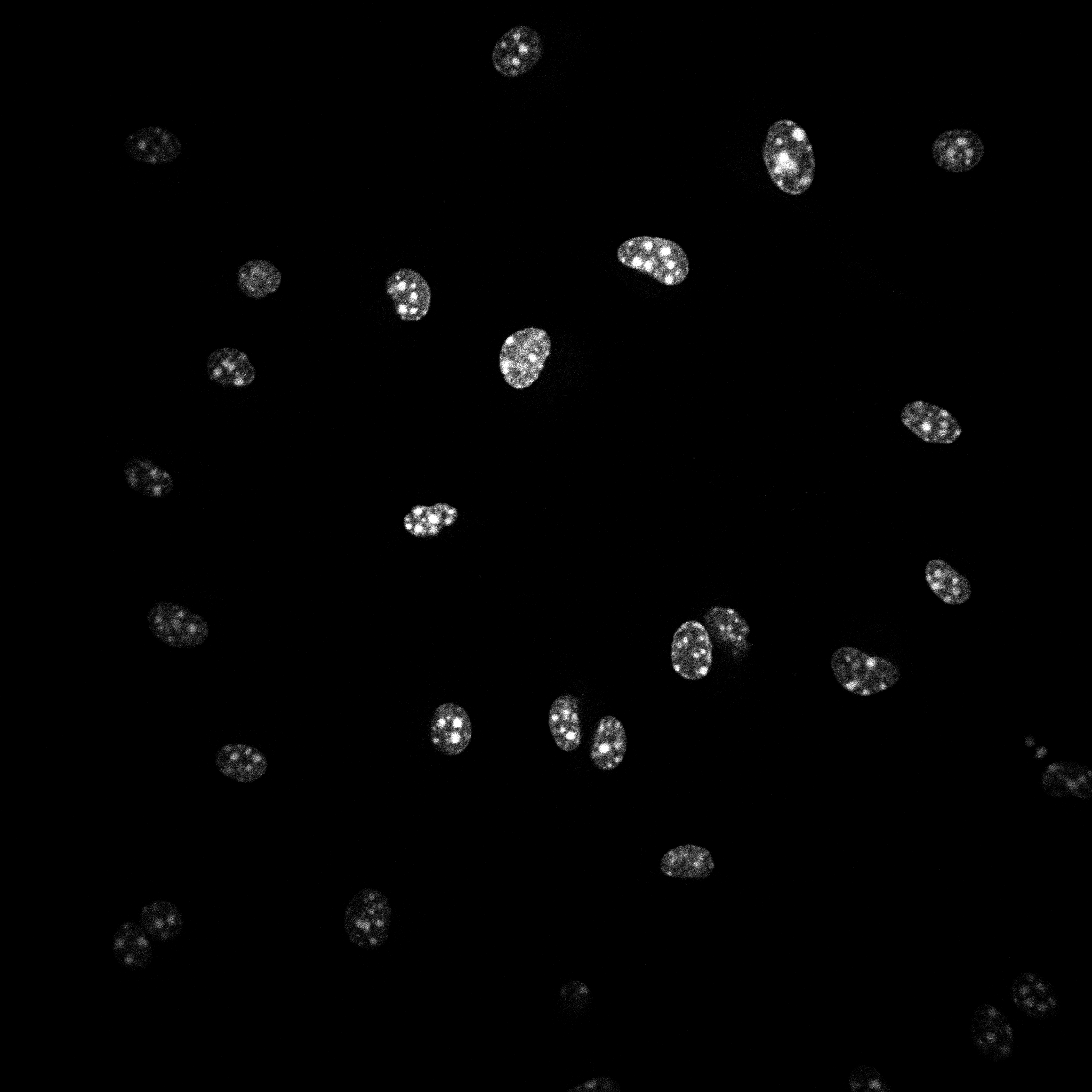

Supplement: Supplementary file 9 — Source Data Fig. 4 [file 44319_2024_117_MOESM9_ESM.zip › Figure 4/4G/4G WT PMAstim DAPI VTIA1B TNFa.tif]

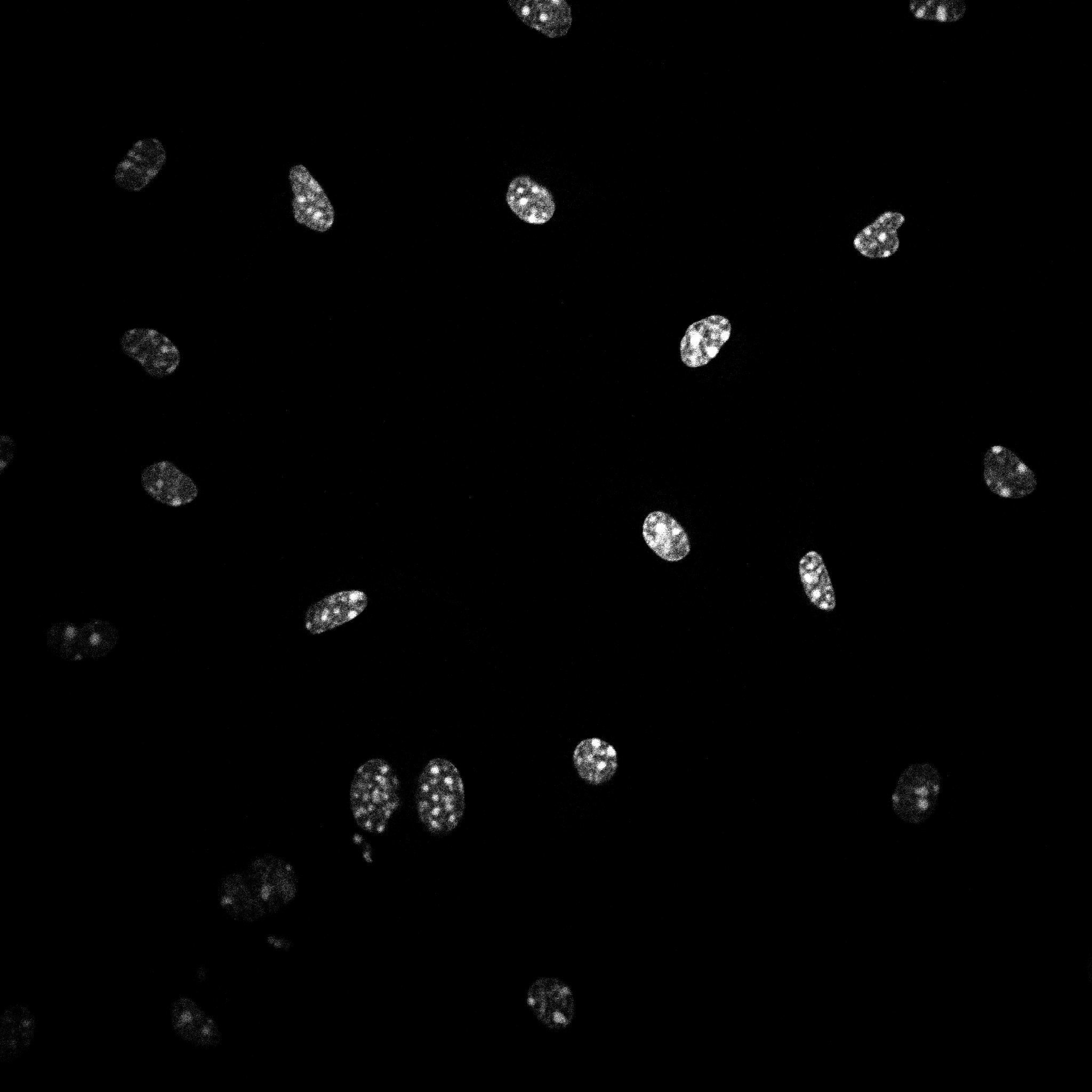

Supplement: Supplementary file 9 — Source Data Fig. 4 [file 44319_2024_117_MOESM9_ESM.zip › Figure 4/4G/4G SORLAKO PMAstim DAPI RAB11 TNFa.tif]

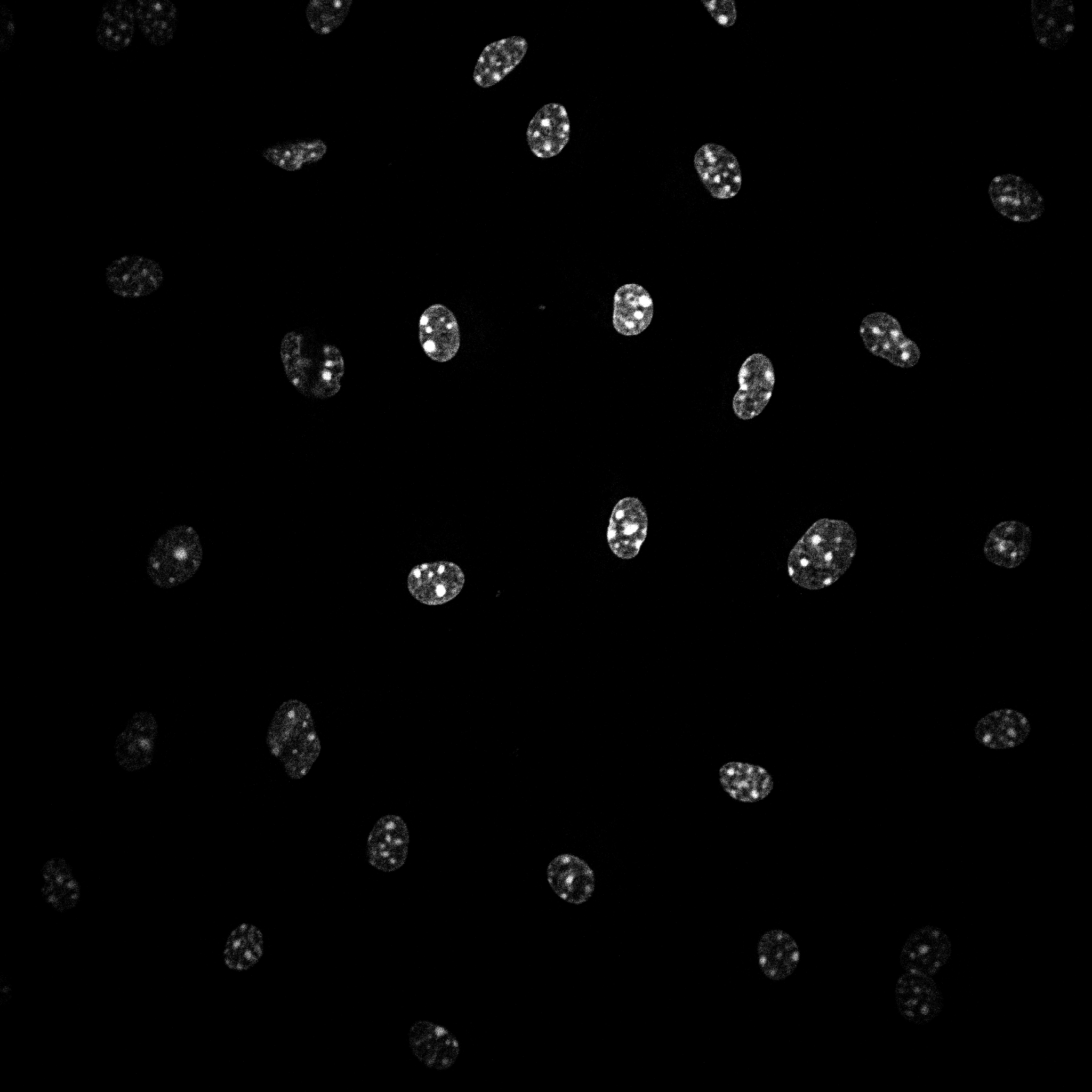

Supplement: Supplementary file 9 — Source Data Fig. 4 [file 44319_2024_117_MOESM9_ESM.zip › Figure 4/4G/4G SORLAKO PMAstim DAPI VTIA1B TNF.tif]

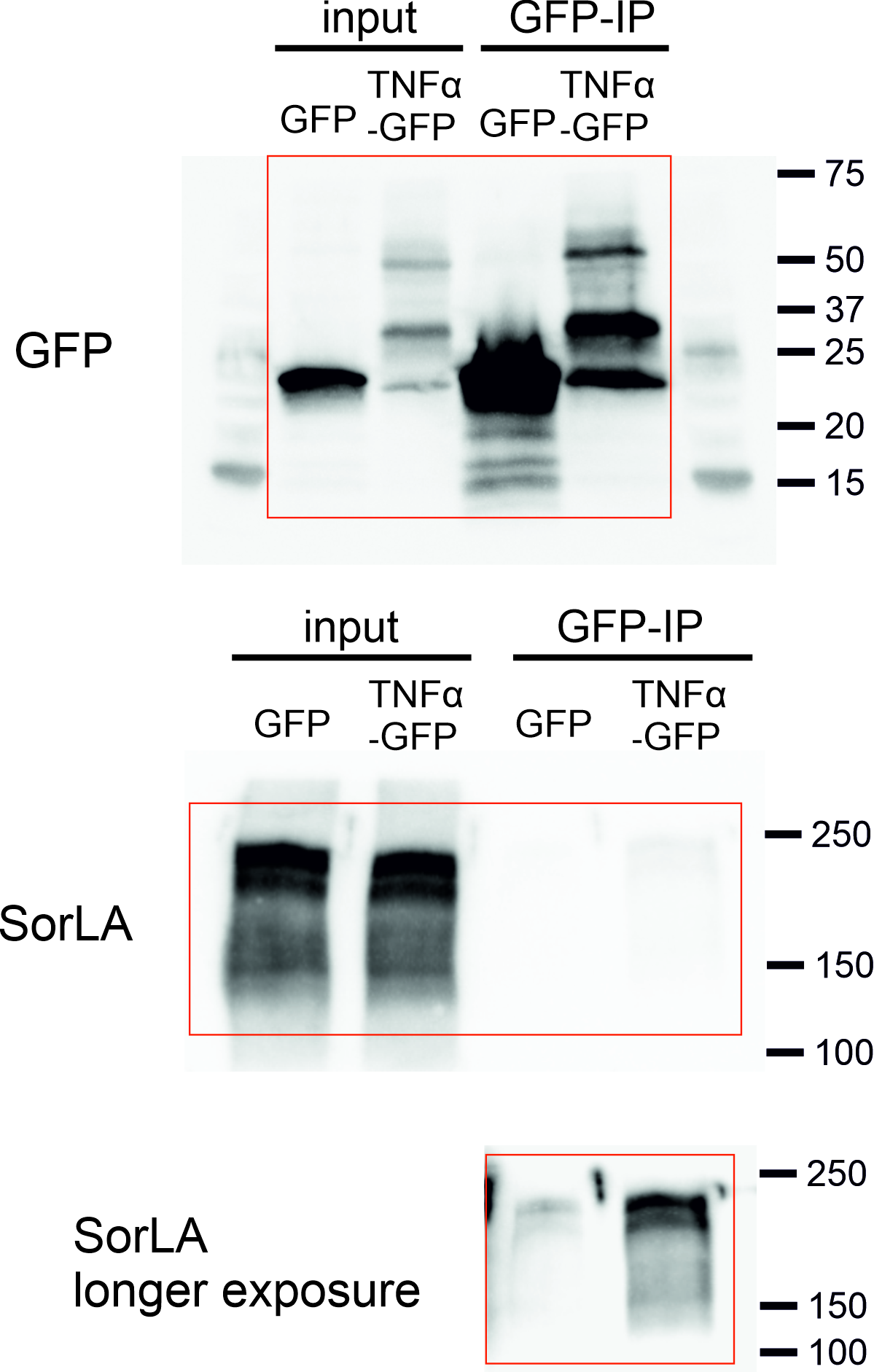

Supplement: Supplementary file 9 — Source Data Fig. 4 [file 44319_2024_117_MOESM9_ESM.zip › Figure 4/4C/4C coIP SorLA TNFa-GFP blots .tif]

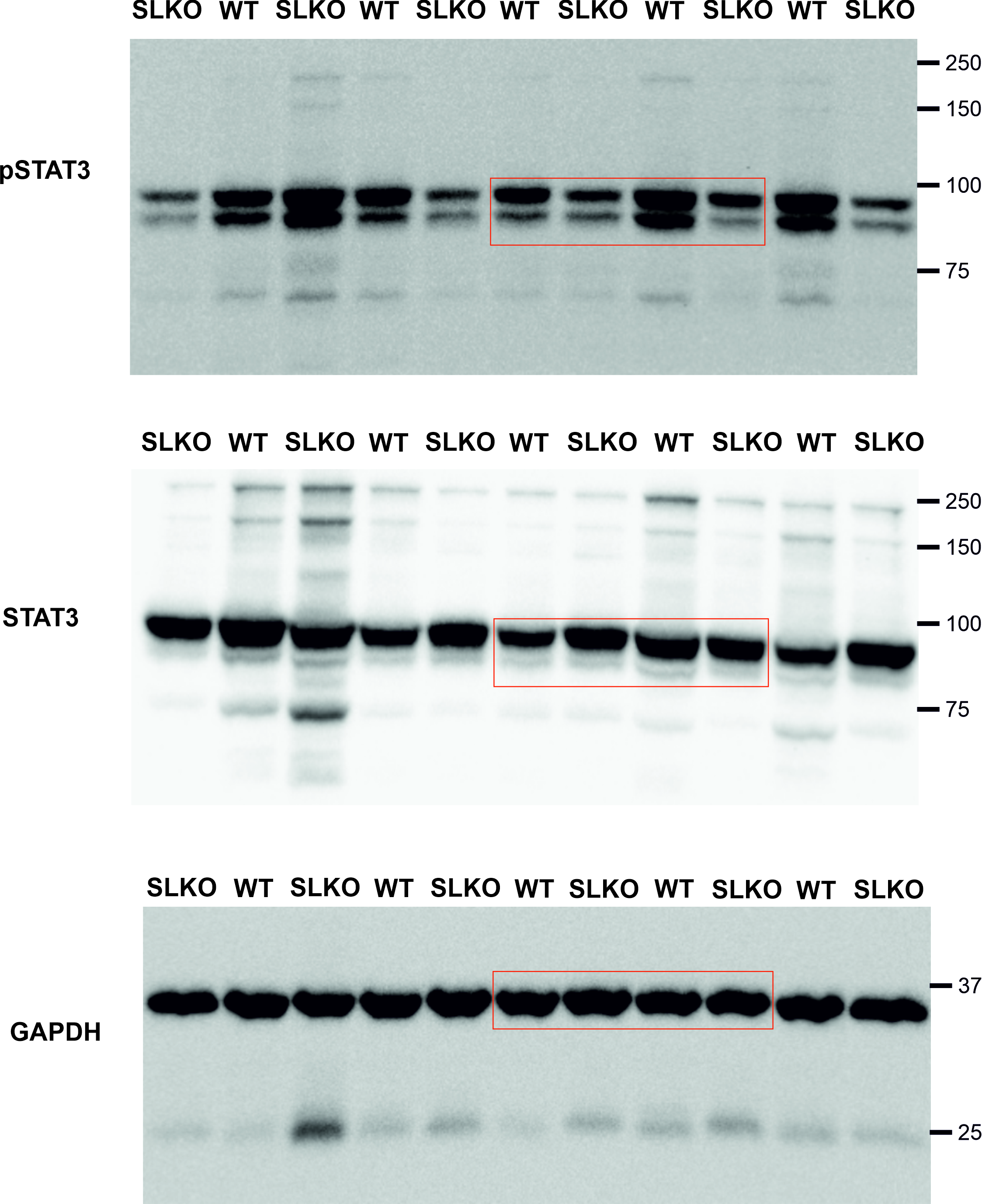

Supplement: Supplementary file 10 — Source Data Fig. 5 [file 44319_2024_117_MOESM10_ESM.zip › Figure 5/5E/5E pSTAT3 blots .tif]

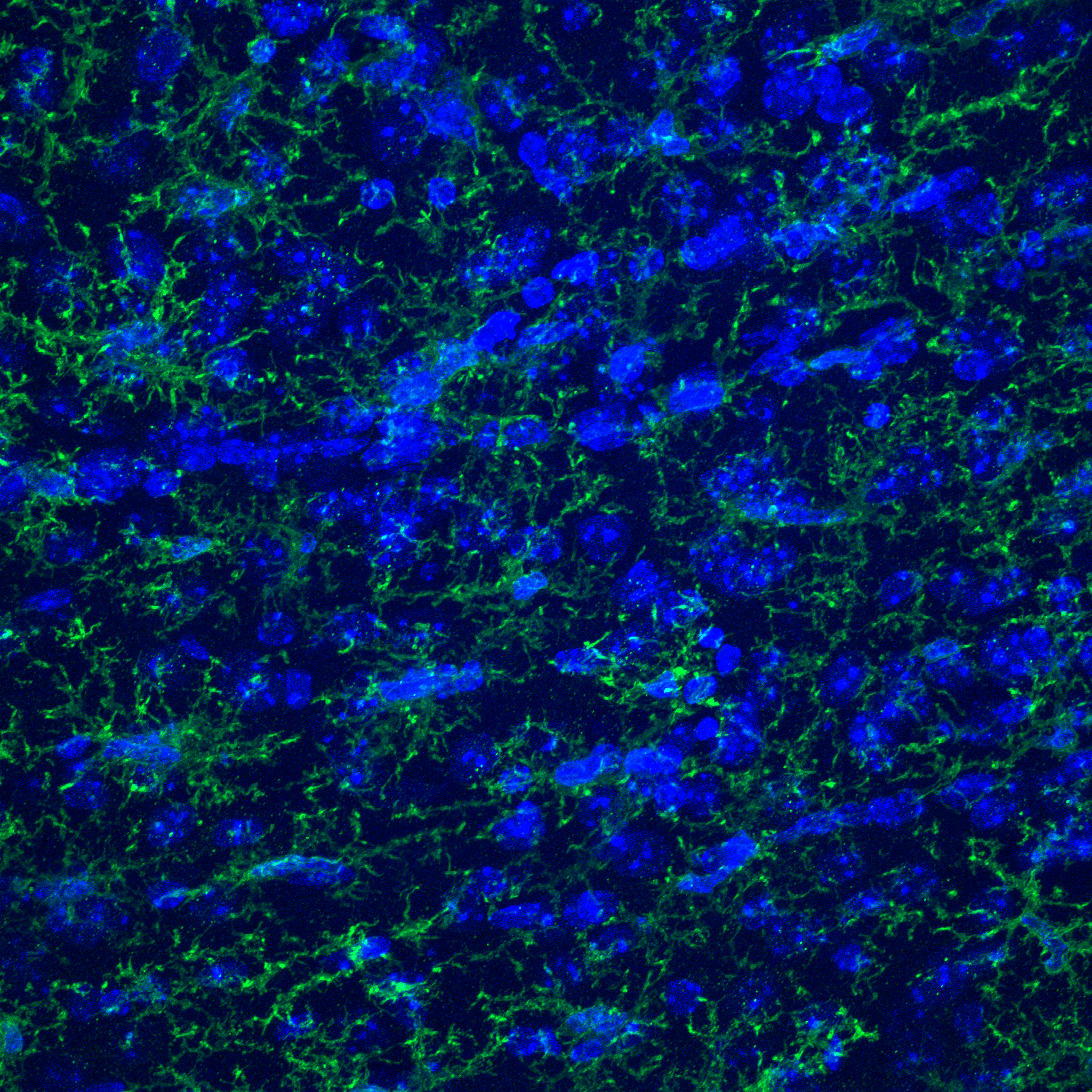

Supplement: Supplementary file 10 — Source Data Fig. 5 [file 44319_2024_117_MOESM10_ESM.zip › Figure 5/5C/5C Tmem119 DAPI max intensity ipsilateral hemisphere.tif]

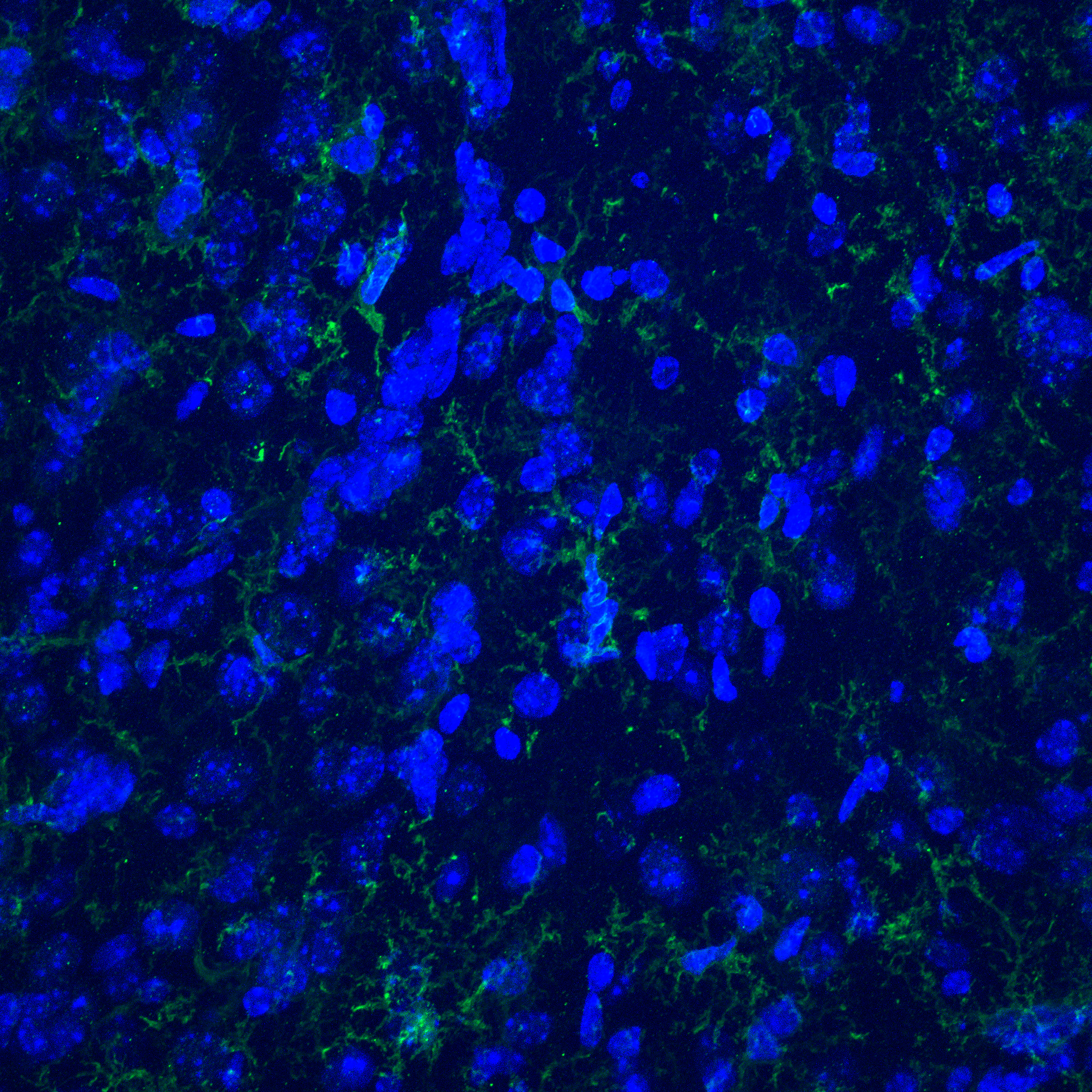

Supplement: Supplementary file 10 — Source Data Fig. 5 [file 44319_2024_117_MOESM10_ESM.zip › Figure 5/5C/5C SLKO Tmem119 max intensity ipsilateral hemisphere.tif]

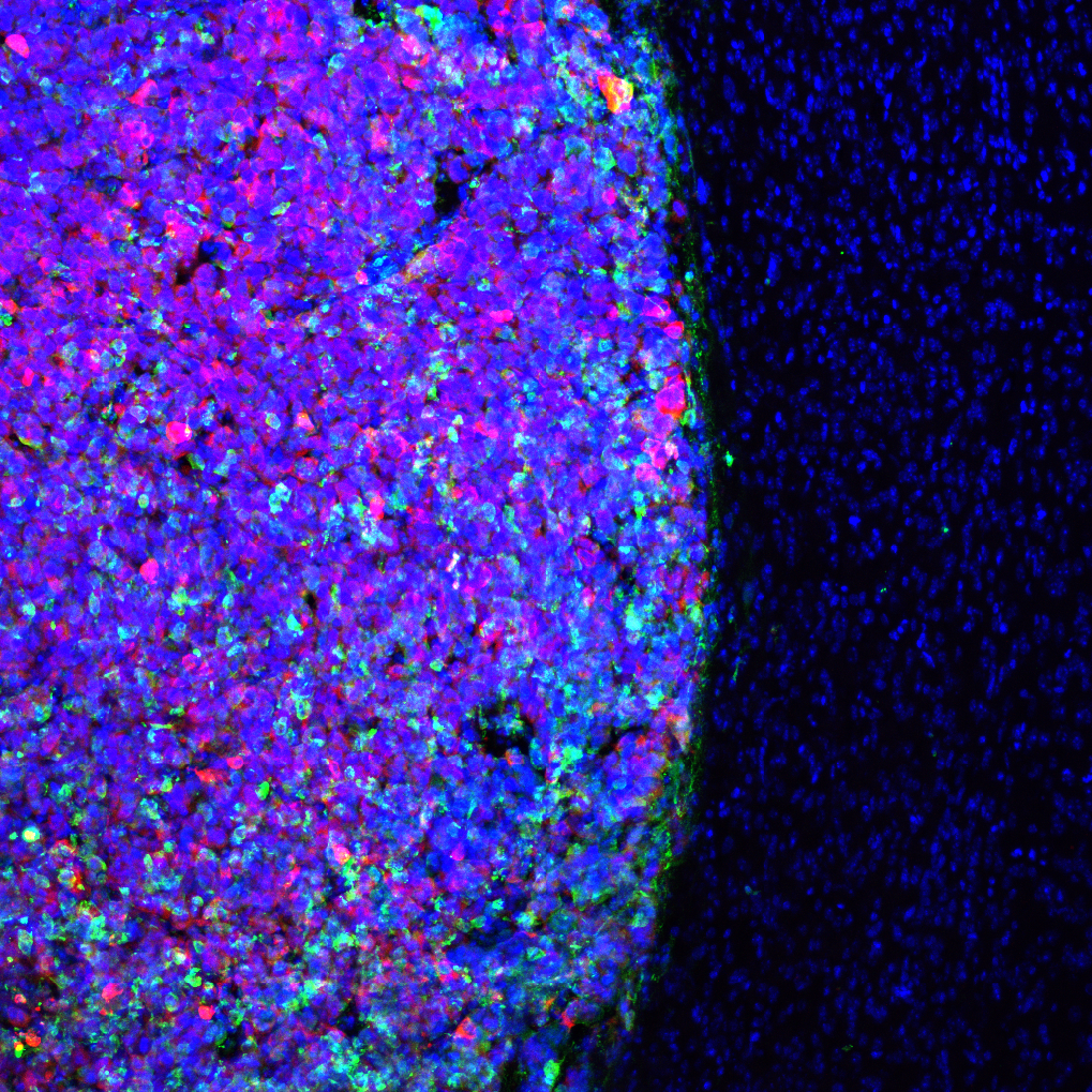

Supplement: Supplementary file 11 — Source Data Fig. 6 [file 44319_2024_117_MOESM11_ESM.zip › Figure 6/6A/6A SLKO DAPI GAL3 TUMOR.tif]

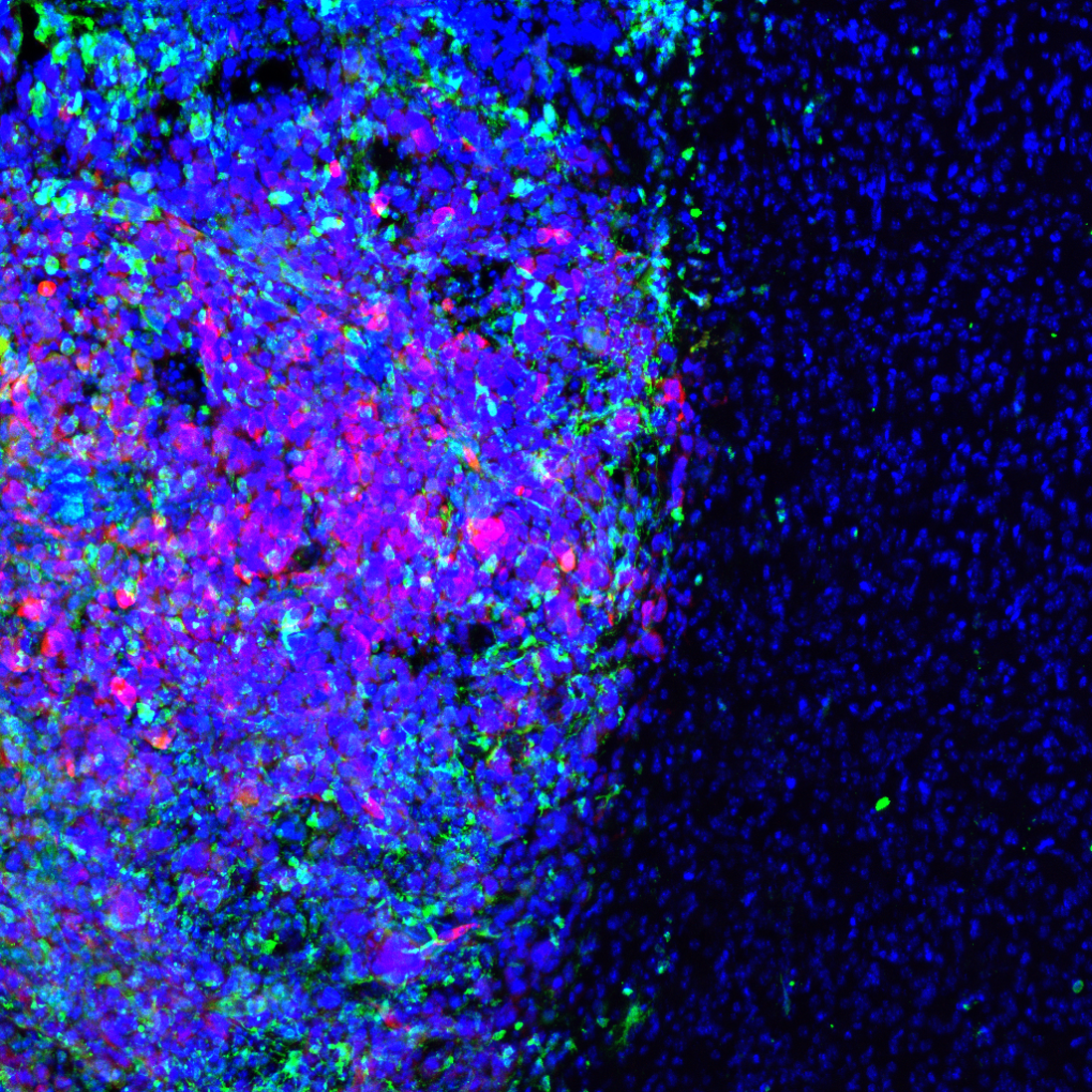

Supplement: Supplementary file 11 — Source Data Fig. 6 [file 44319_2024_117_MOESM11_ESM.zip › Figure 6/6A/6A WT DAPI GAL3 TUMOR.tif]

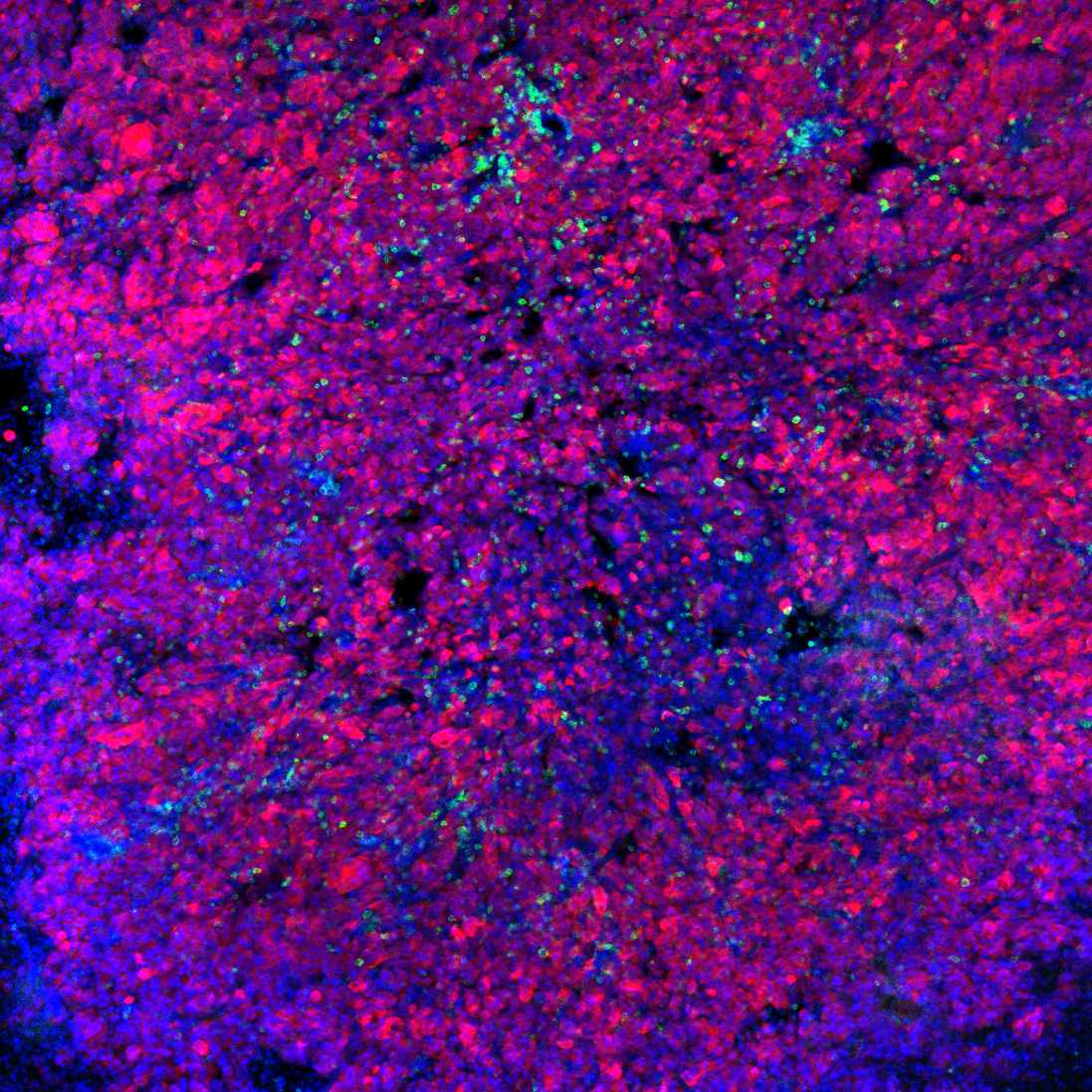

Supplement: Supplementary file 11 — Source Data Fig. 6 [file 44319_2024_117_MOESM11_ESM.zip › Figure 6/6B/6B SLKO DAPI CD8 TUMOR.tif]

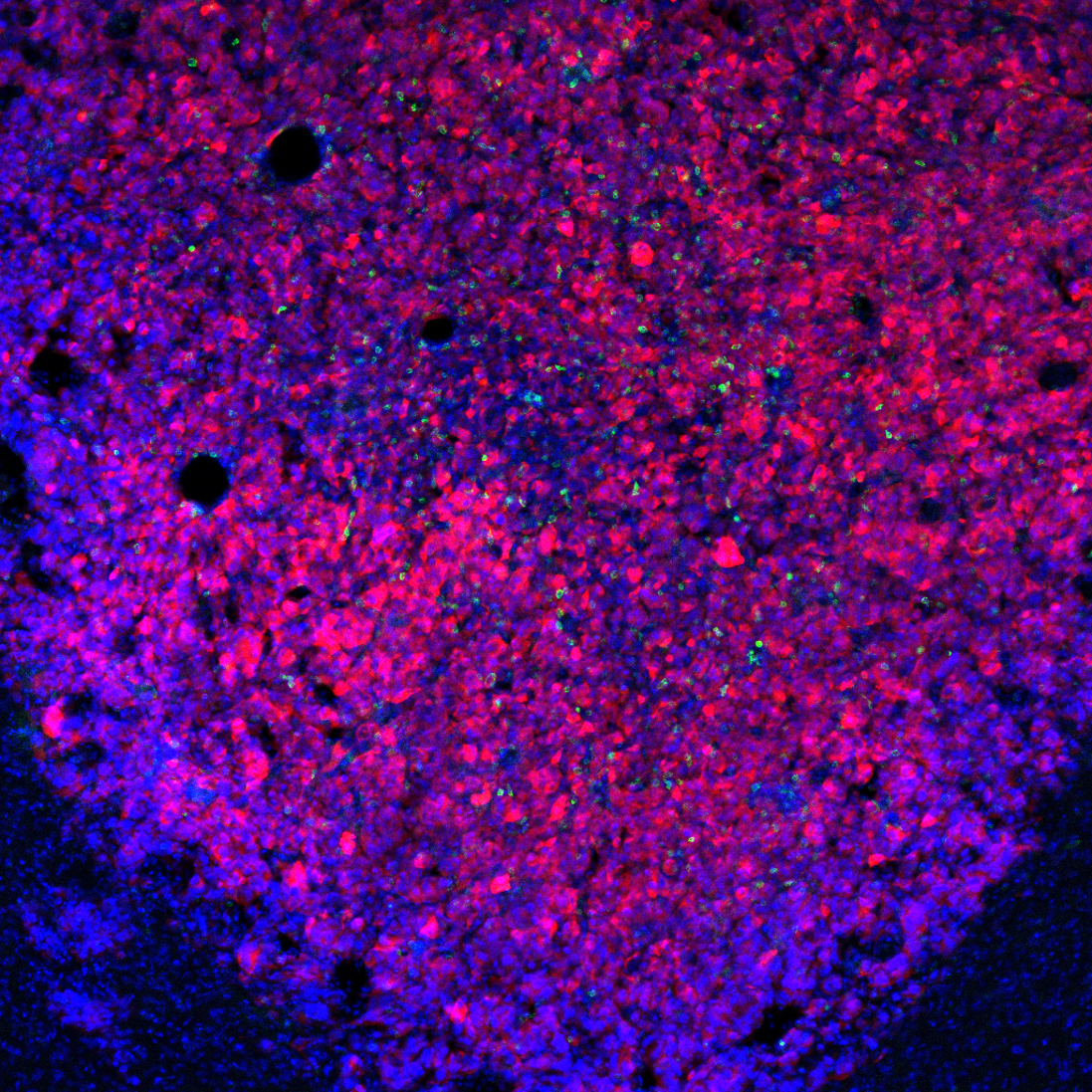

Supplement: Supplementary file 11 — Source Data Fig. 6 [file 44319_2024_117_MOESM11_ESM.zip › Figure 6/6B/6B WT DAPI CD8 TUMOR.tif]

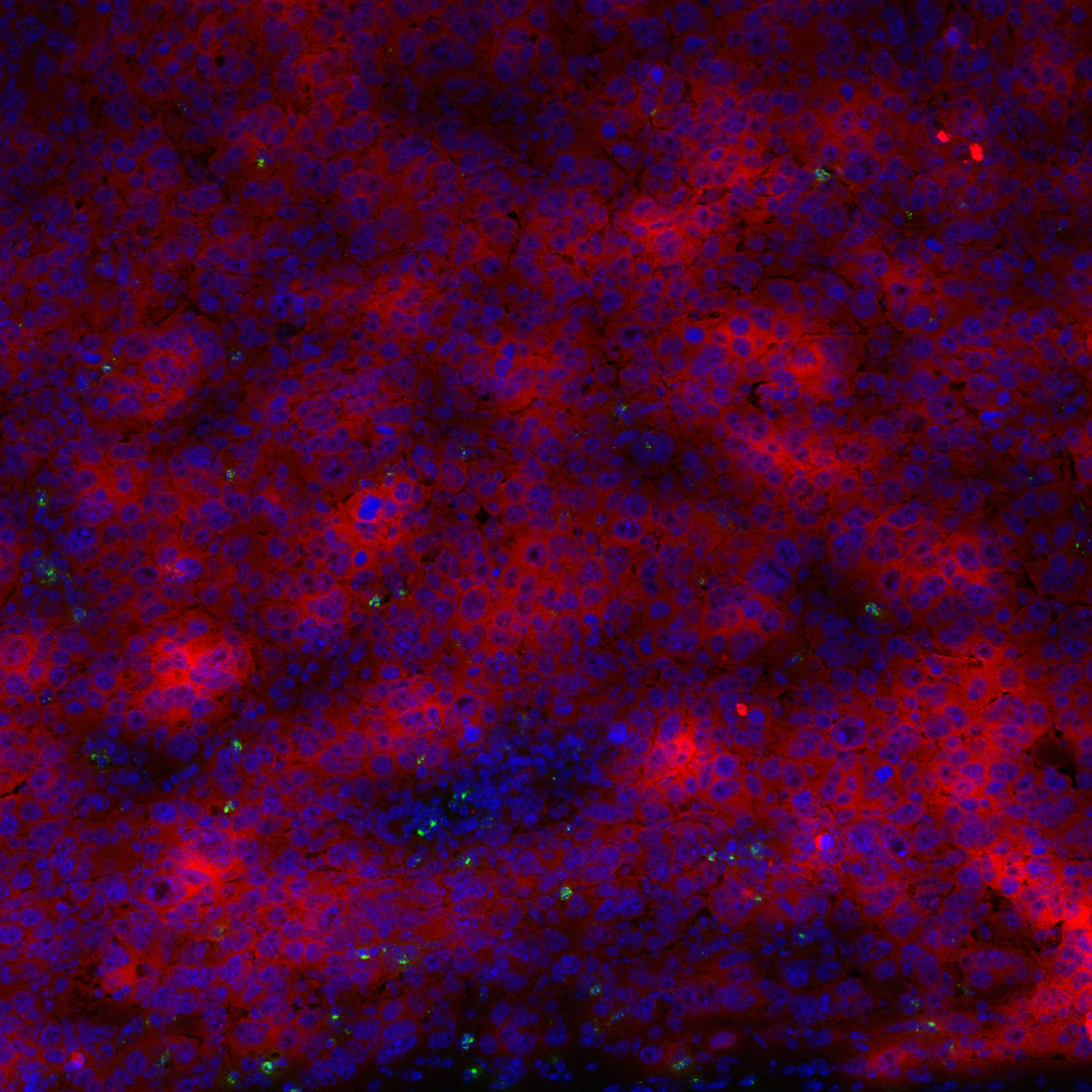

Supplement: Supplementary file 11 — Source Data Fig. 6 [file 44319_2024_117_MOESM11_ESM.zip › Figure 6/6E/6E WT DAPI MPO TUMOR.tif]

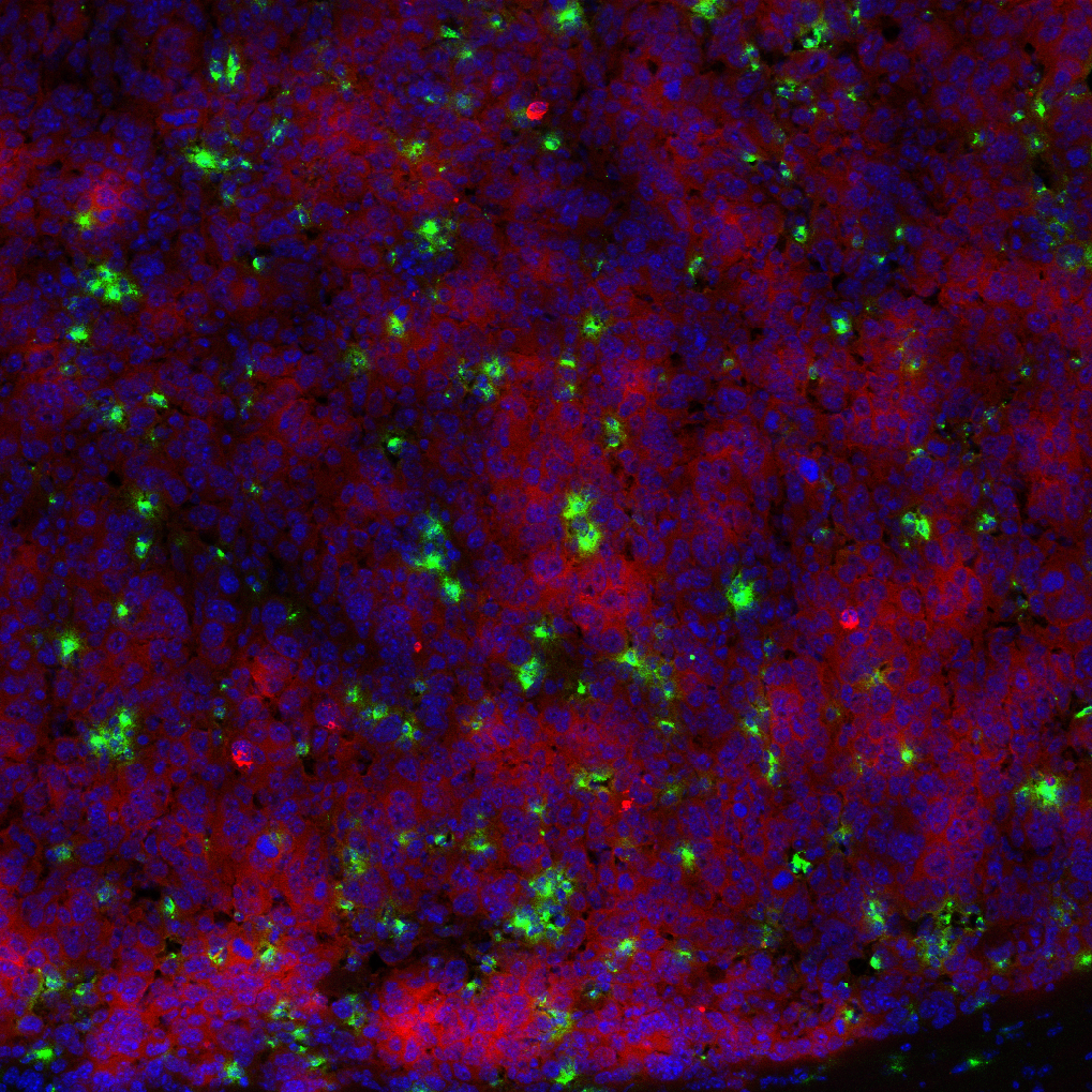

Supplement: Supplementary file 11 — Source Data Fig. 6 [file 44319_2024_117_MOESM11_ESM.zip › Figure 6/6E/6E SLKO DAPI MPO TUMOR_.tif]

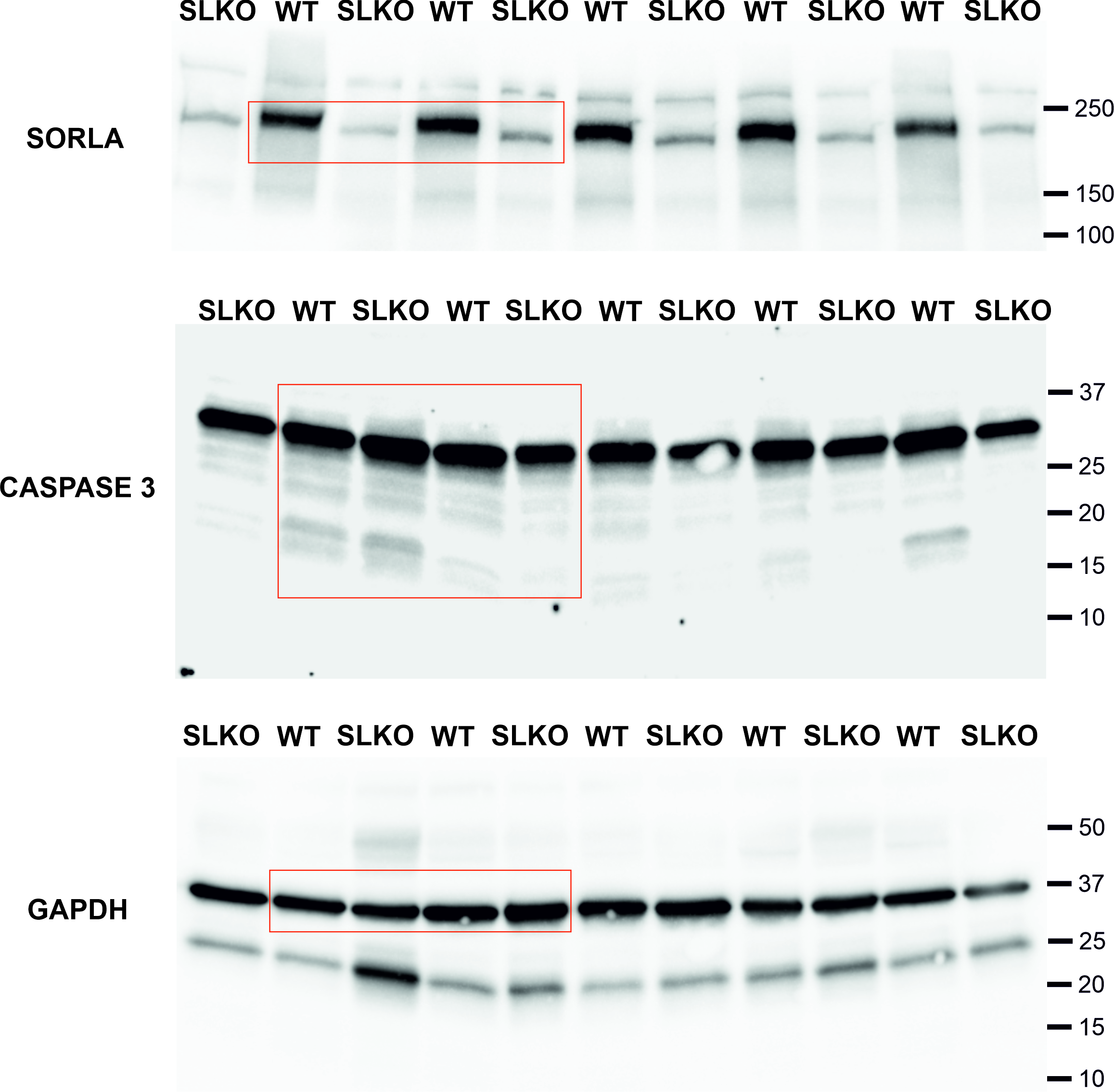

Supplement: Supplementary file 12 — Source Data Fig. 7 [file 44319_2024_117_MOESM12_ESM.zip › Figure 7/7B/7B SorLA_Casp3 blots.tif]

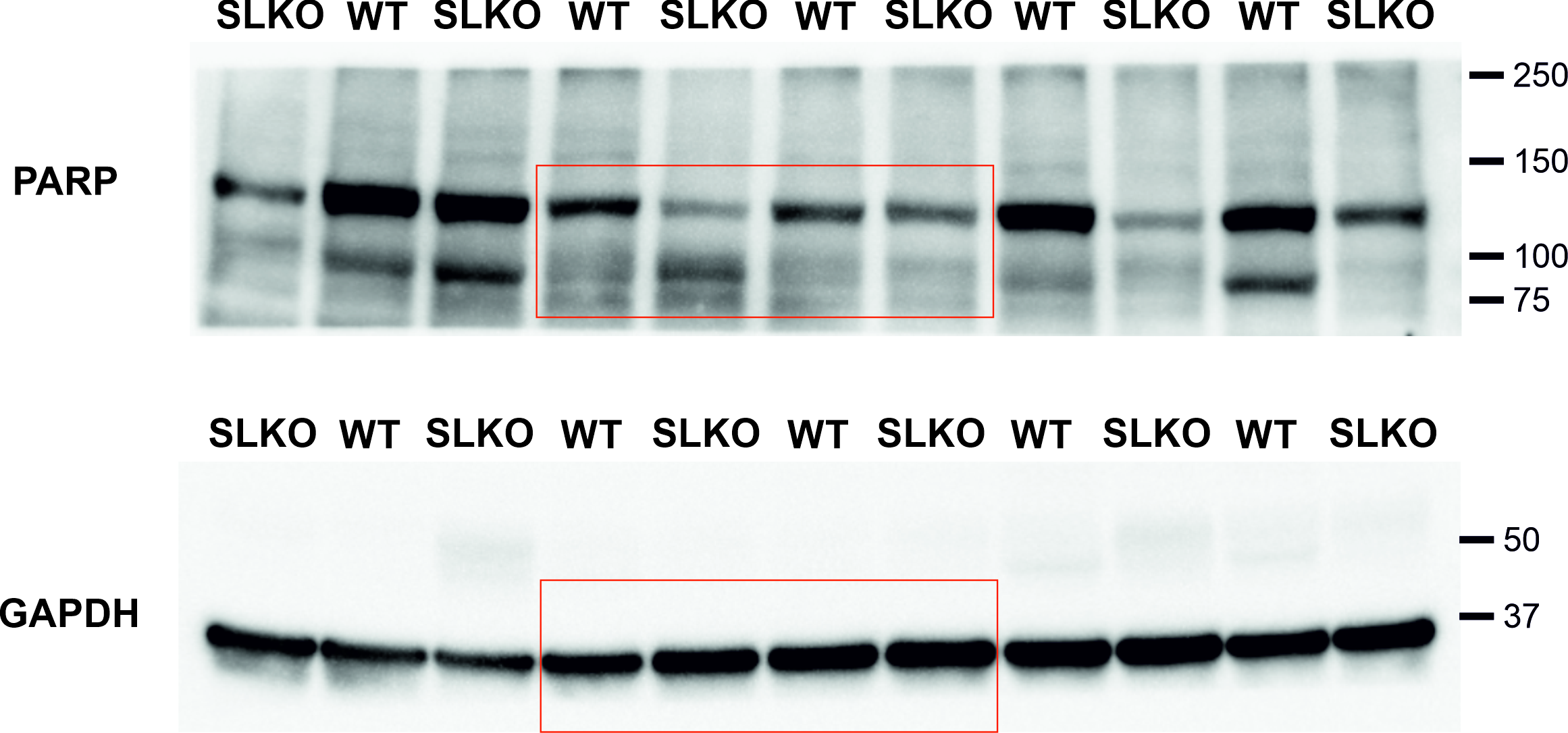

Supplement: Supplementary file 12 — Source Data Fig. 7 [file 44319_2024_117_MOESM12_ESM.zip › Figure 7/7A/7A PARP blots.tif]

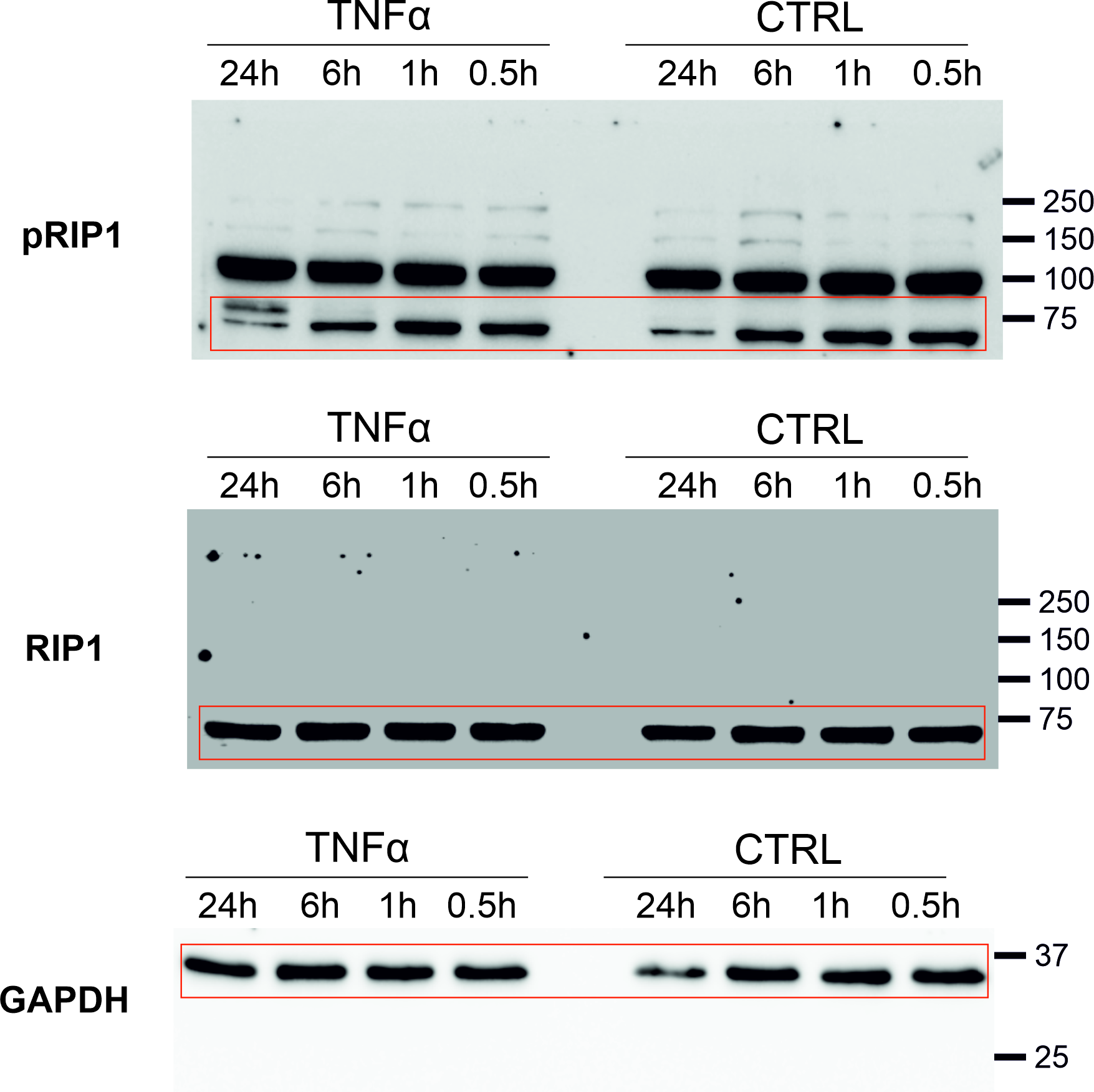

Supplement: Supplementary file 12 — Source Data Fig. 7 [file 44319_2024_117_MOESM12_ESM.zip › Figure 7/7F/7F pRIP1 blots .tif]

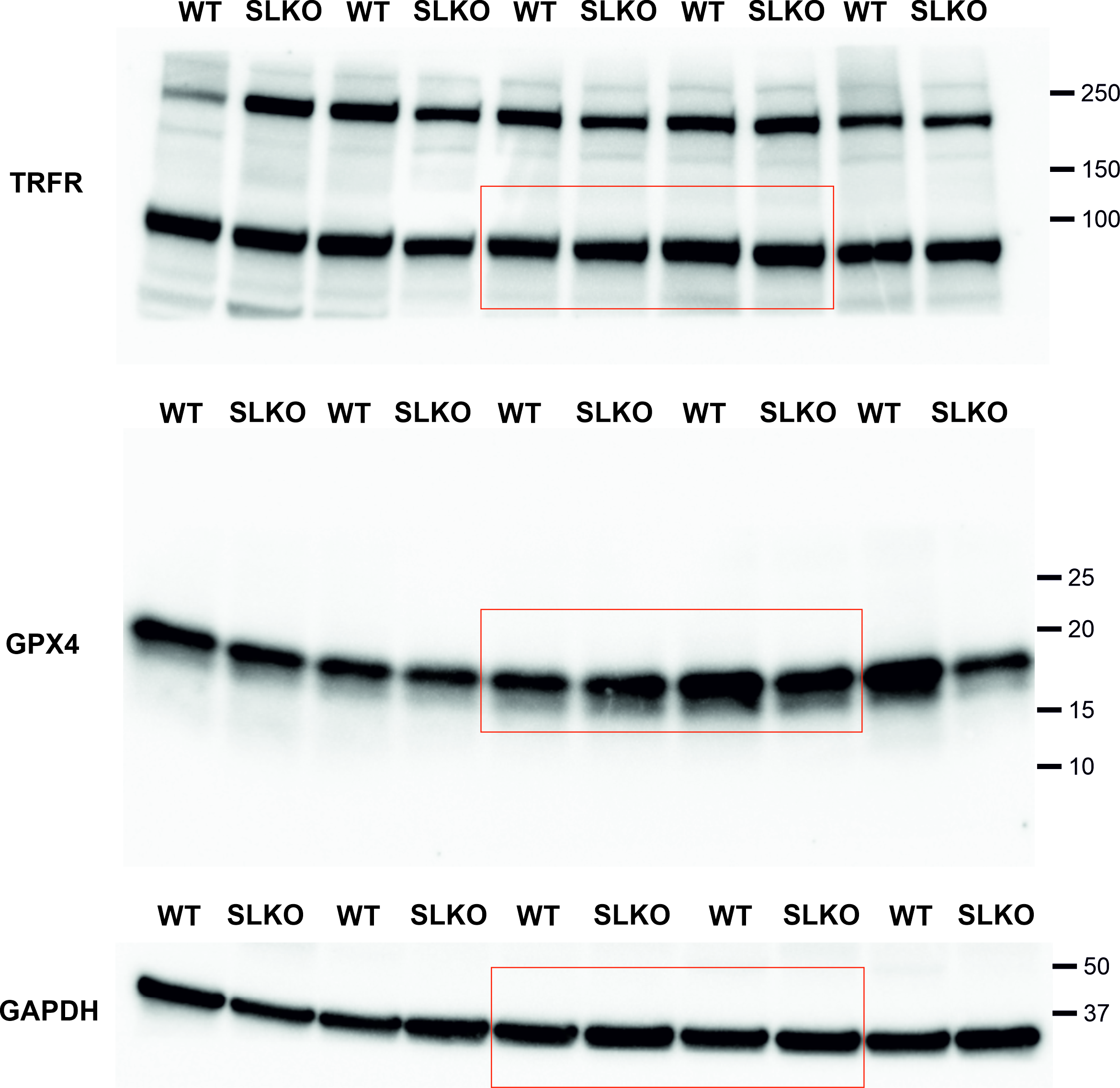

Supplement: Supplementary file 12 — Source Data Fig. 7 [file 44319_2024_117_MOESM12_ESM.zip › Figure 7/7C/7C TRFR GPX4 blots.tif]

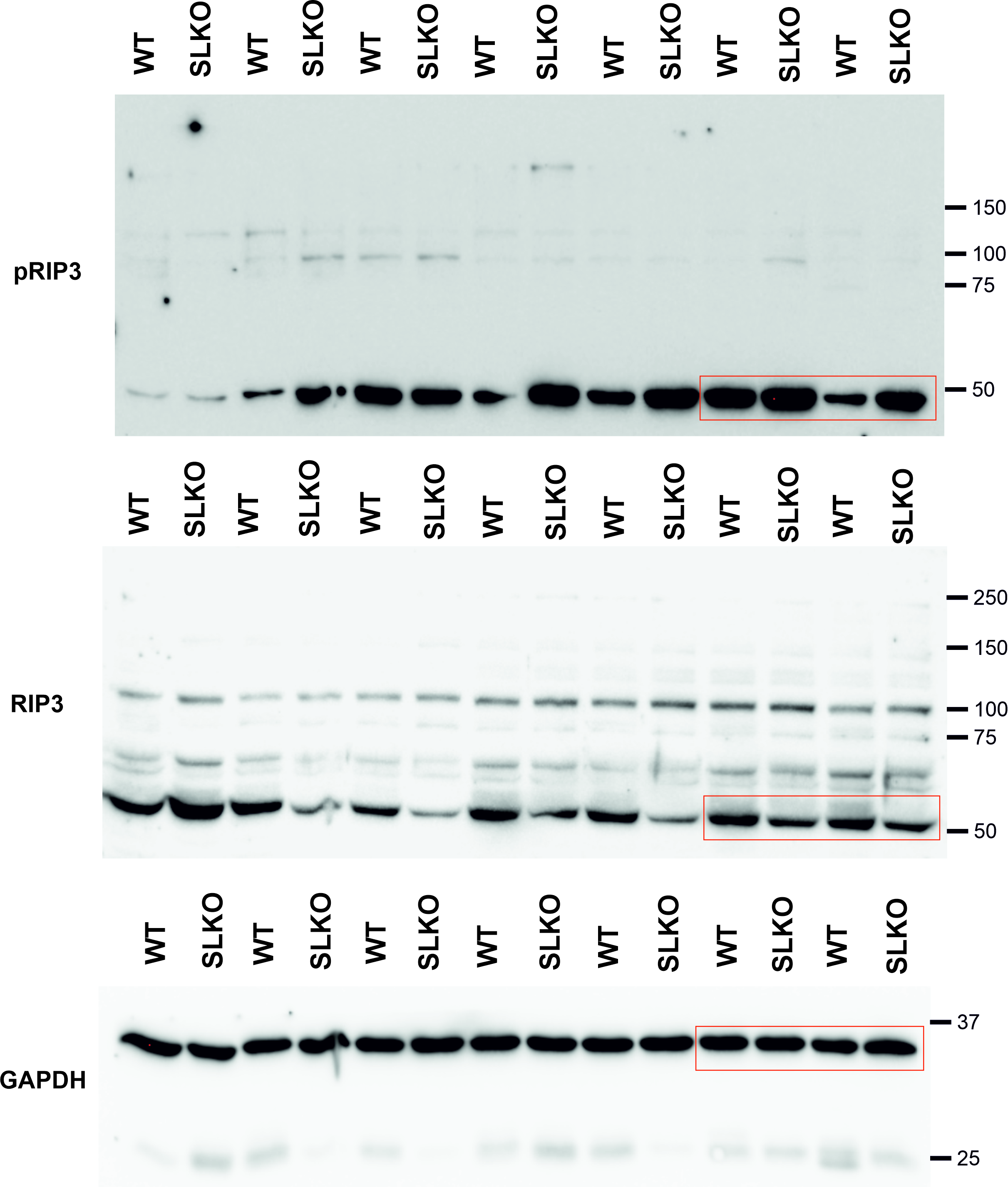

Supplement: Supplementary file 12 — Source Data Fig. 7 [file 44319_2024_117_MOESM12_ESM.zip › Figure 7/7E/7E pRIP3 blots.tif]

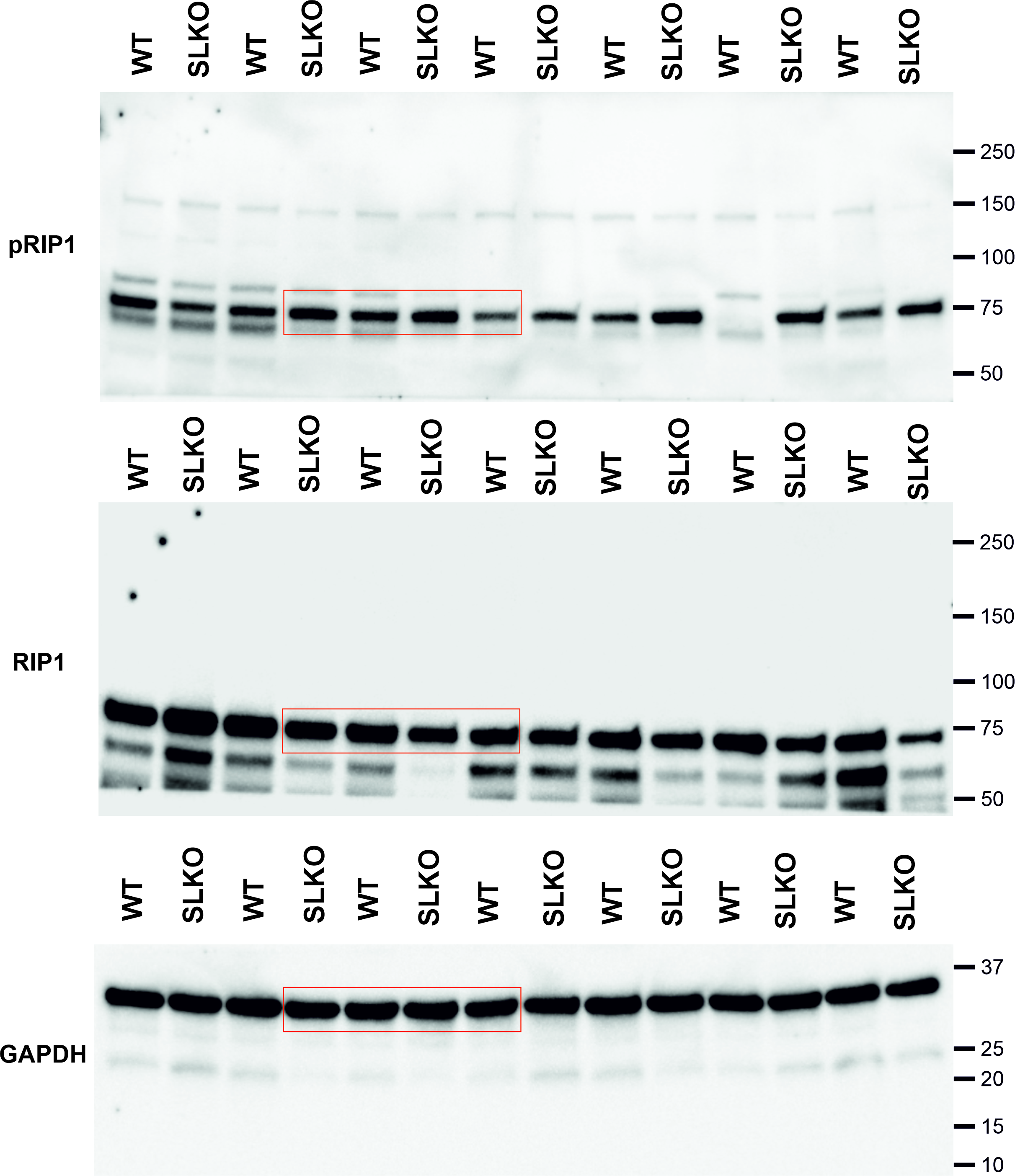

Supplement: Supplementary file 12 — Source Data Fig. 7 [file 44319_2024_117_MOESM12_ESM.zip › Figure 7/7D/7D pRIP1 blots.tif]
