# Supplementary material for: SorLA restricts TNFα release from microglia to shape a glioma-supportive brain microenvironment
Source: EMBO Rep. 2024 Mar 18;25(5):13. doi: 10.1038/s44319-024-00117-6 (PMC11094098; doi:10.1038/s44319-024-00117-6)
Supplement: Supplementary file 10 — Source Data Fig. 5 [file 44319_2024_117_MOESM10_ESM.zip › Figure 5/5C/5C README .docx]

**WT Tmem119 DAPI max intensity ipsilateral hemisphere**

The picture was split into two separate channels: DAPI and Tmem119 in ImageJ software. DAPI staining was not included in the analysis from the very beginning. Z-stacks no. 12 to no. 67 from Tmem119 channel were duplicated and max intensity projection was performed. Then the picture was flipped vertically and saved as a new file. The intensity of Tmem119 staining (green) was adjusted in Imaris 9.1.2. Software afterwards.

**SLKO Tmem119 DAPI max intensity ipsilateral hemisphere**

The picture was split into two separate channels: DAPI and Tmem119 in ImageJ software. DAPI staining was not included in the analysis from the very beginning. Z-stacks no. 12 to no. 29 were duplicated and max intensity projection was performed. Then the picture was flipped vertically and saved as a new file. The intensity of Tmem119 staining (green) was adjusted in Imaris 9.1.2. Software afterwards.
