# Supplementary material for: SorLA restricts TNFα release from microglia to shape a glioma-supportive brain microenvironment
Source: EMBO Rep. 2024 Mar 18;25(5):13. doi: 10.1038/s44319-024-00117-6 (PMC11094098; doi:10.1038/s44319-024-00117-6)
Supplement: Supplementary file 11 — Source Data Fig. 6 [file 44319_2024_117_MOESM11_ESM.zip › Figure 6/6E/6E README.docx]

Confocal images for MPO staining were mirrored horizontally prior to placing on the figure.
