# Supplementary material for: SorLA restricts TNFα release from microglia to shape a glioma-supportive brain microenvironment
Source: EMBO Rep. 2024 Mar 18;25(5):13. doi: 10.1038/s44319-024-00117-6 (PMC11094098; doi:10.1038/s44319-024-00117-6)
Supplement: Supplementary file 12 — Source Data Fig. 7 [file 44319_2024_117_MOESM12_ESM.zip › Figure 7/7F/7F README.docx]

After cropping, blots were flipped horizontally prior to placing them on the figure.
