# Supplementary material for: SorLA restricts TNFα release from microglia to shape a glioma-supportive brain microenvironment
Source: EMBO Rep. 2024 Mar 18;25(5):13. doi: 10.1038/s44319-024-00117-6 (PMC11094098; doi:10.1038/s44319-024-00117-6)
Supplement: Supplementary file 14 — Expanded View Figures [file 44319_2024_117_MOESM14_ESM.pdf]

Expanded View Figures

Figure EV1. *SORL1* is expressed in GAMs.

(**A**) UMAP projection of single cells from human ndGBM tumors described in Abdelfattah et al, grouped in 21 clusters. Clusters identified as GAMs are indicated with purple dashed line. (**B**, **C**) UMAP projections presenting expression of *SORL1* (**B**) and GAMs marker genes (**C**) in all clusters as exemplified in (**A**). Expression levels are normalized with SCT. (**D**) UMAP projection of single cells from human GBM tumors described in Neftel et al, grouped in 21 clusters. Cluster identified as GAMs is indicated with purple dashed line. (**E**, **F**) UMAP projections presenting expression of *SORL1* (**E**) and GAMs marker genes (**F**) in all clusters as exemplified in (**D**). Expression levels are normalized with SCT.

 © The Author(s)

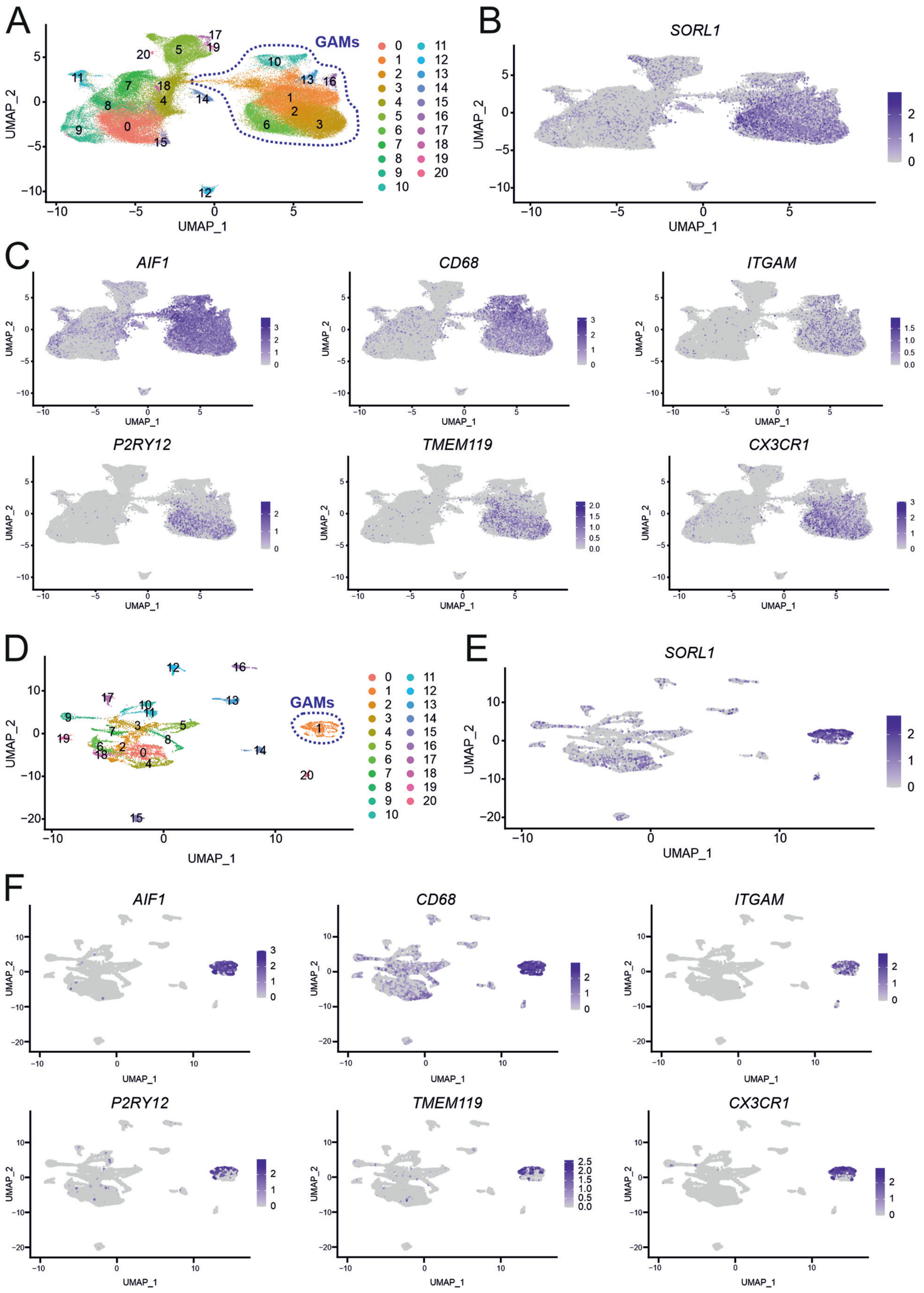

A
B
C
D
E
F
GAMs
SORL1
AIF1
CD68
ITGAM
P2RY12
TMEM119
CX3CR1
UMAP_1
UMAP_2

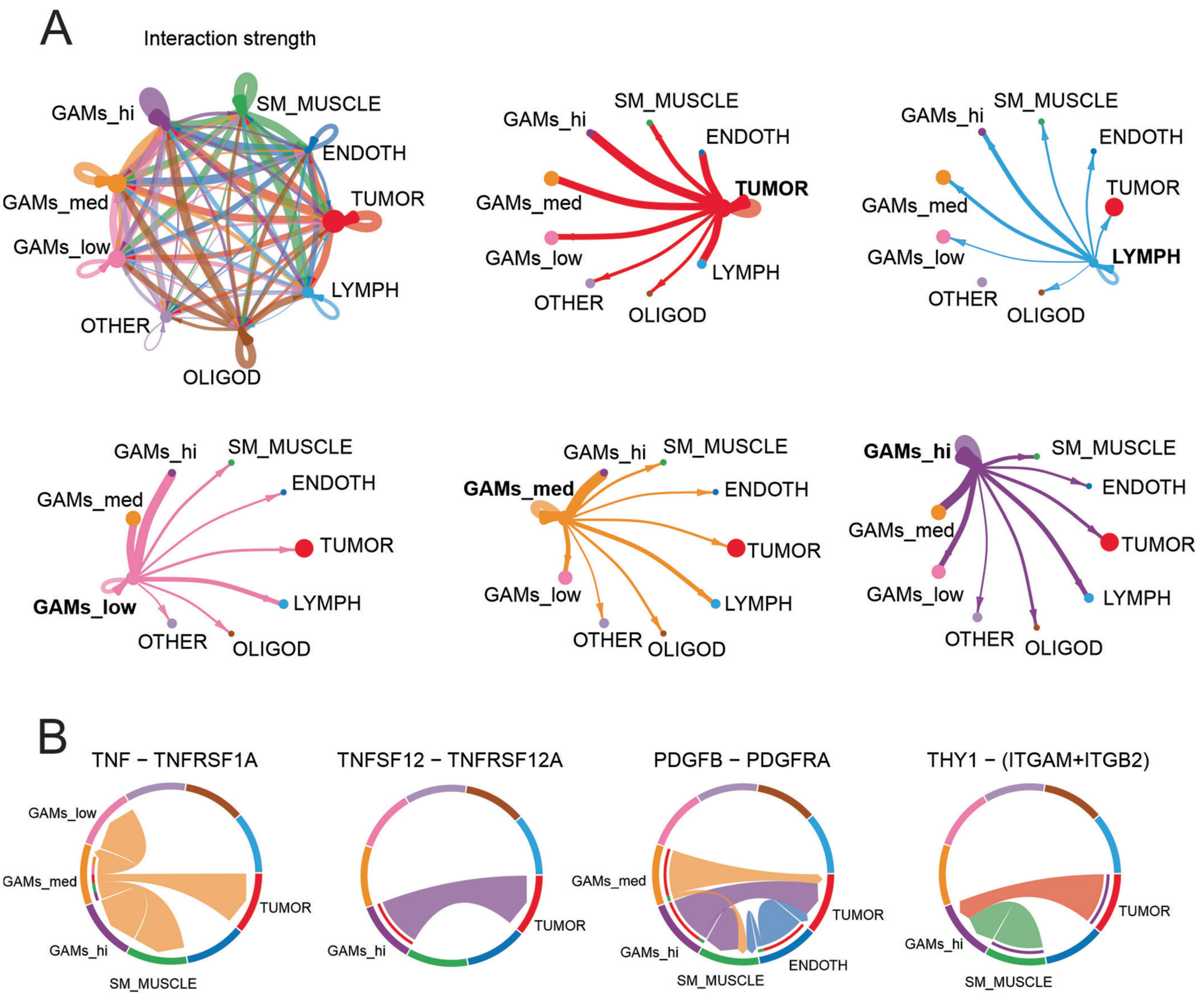

Figure EV2. Interaction networks between cell populations in newly diagnosed GBM tumors.

(**A**) Network plots showing strength of ligand-receptor interactions between cell populations. The line width is proportional to the number of ligand-receptor pairs identified. (**B**) Chord diagrams indicating selected ligand-receptor pairs mediating interaction between cell populations. Width of chords is proportional to signal strength of the given ligand-receptor pair.

 © The Author(s)

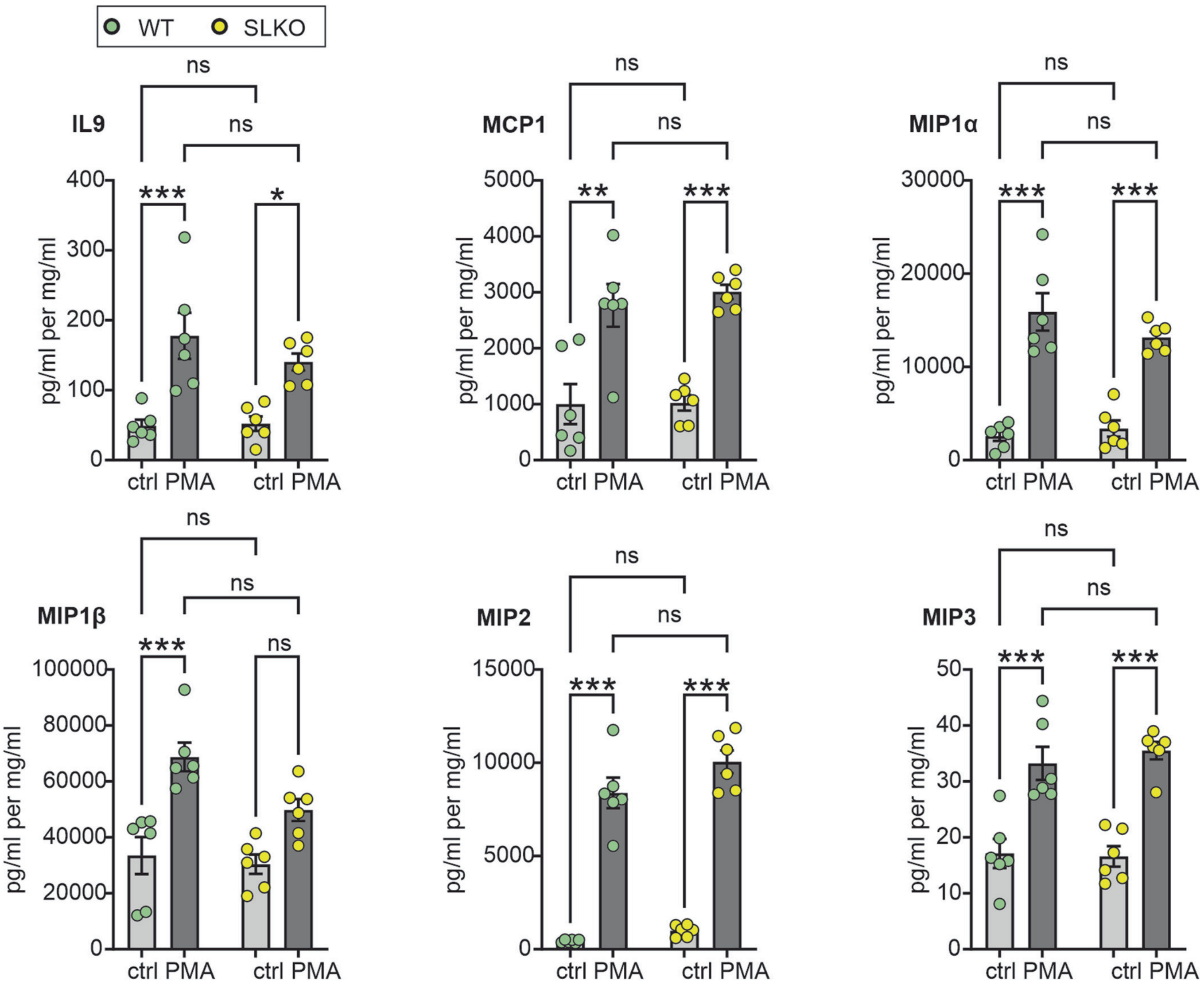

Figure EV3. SorLA deficiency has no major impact on microglial cytokine secretion.

Cytokine levels as determined by ELISA in cell culture medium from primary WT and SorLA-KO microglia either untreated (ctrl) or treated with PMA for 24 h. Cytokine levels were normalized to the protein content in the respective cell lysates. $n = 6$ biological replicates. Data information: data are presented as mean ± SEM. ns not significant; *$P < 0.05$; **$P < 0.01$; ***$P < 0.001$ in two-way ANOVA with Tukey's multiple comparisons test.

© The Author(s)

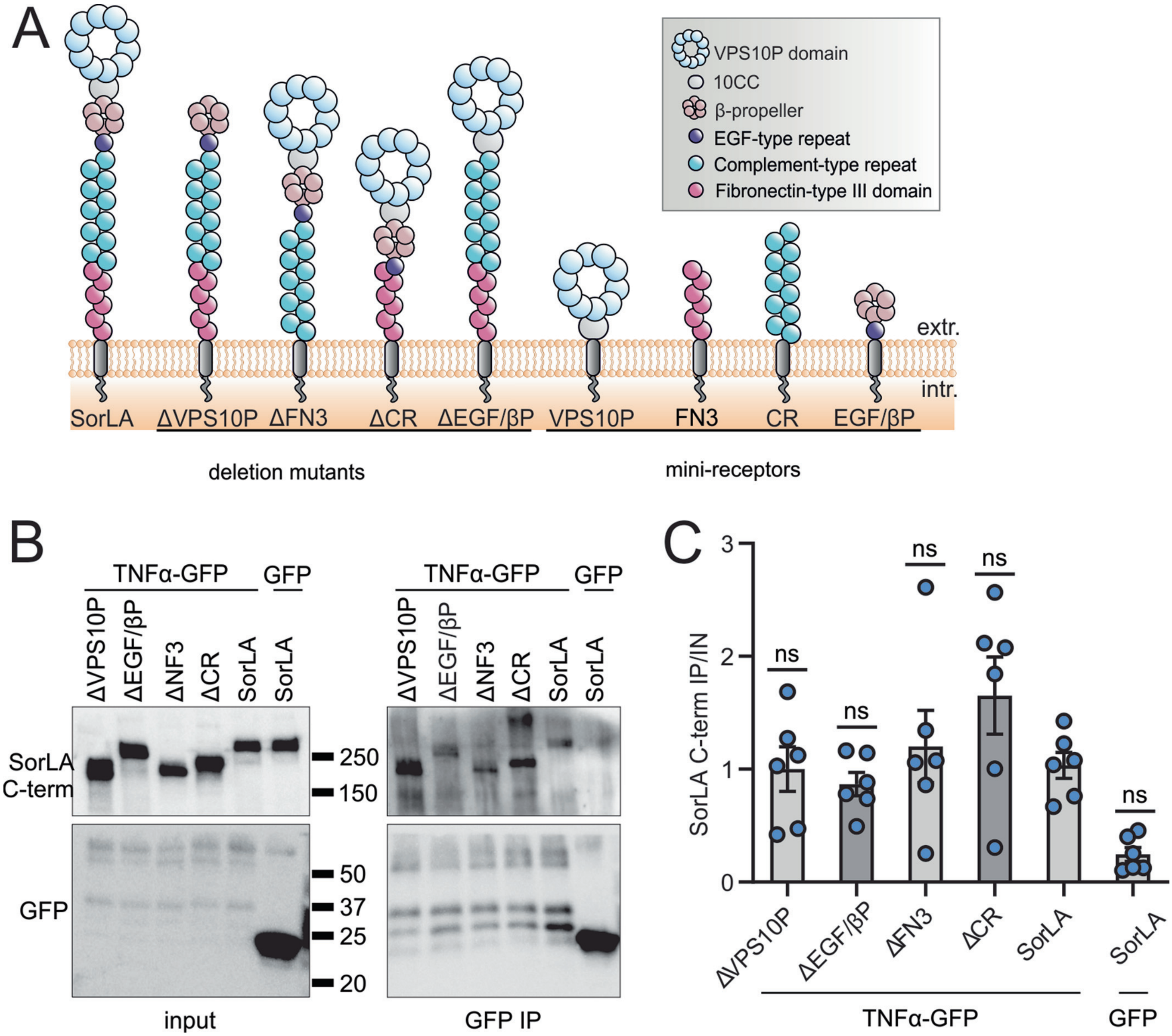

Figure EV4. Binding of SorLA mutants to TNFα.

(**A**) Schematic representation of SorLA structure and the mutant proteins used in this study. (**B**) Co-immunoprecipitation (co-IP) of SorLA deletion mutants with TNFα-GFP overexpressed in HEK293 cells after GFP-IP. GFP serves as a negative control. SorLA was detected using an antibody raised against its C-terminus. (**C**) Quantification of the results of 6 biological replicates as exemplified in (**B**). Ratio of co-IP and input signals (IP/IN) was calculated for each transfection variant. Data information: (**C**) Data are presented as mean ± SEM. ns not significant in one-way ANOVA with Tukey's multiple comparisons test, comparing to full-length SorLA.

 © The Author(s)
